# Supplementary material for: Interaction between games give rise to the evolution of moral norms of cooperation
Source: PLoS Comput Biol. 2022 Sep 29;18(9):e1010429. doi: 10.1371/journal.pcbi.1010429 (PMC9521931; doi:10.1371/journal.pcbi.1010429)
Supplement: S1 Text — Overview of the models, details of methods and further analysis of the models. (PDF) [file pcbi.1010429.s001.pdf]

Supplemental Information for
Interaction between games give rise to the evolution of moral norms of cooperation

Mohammad Salahshour

Contents

A Overview of the models **2**
A.1 The mixed population 2
A.1.1 Model 1: The direct interaction model 2
A.1.2 Model 2: The reputation-based model. 2
A.2 Structured population 3

B Details of the Methods and derivation of replicator dynamics **3**
B.1 Games and their payoff 3
B.2 Soft and hard strategies 4
B.3 The replicator dynamics 4
B.4 Calculation of the connected correlation functions 6

C Nash equilibria when game B is the Battle of the Sexes and the Leader game **7**

D Supplementary Videos **7**

E Analysis of direct interaction model in a mixed population **8**
E.1 Three archetypal game 8
E.2 Dependence on continuous variation of game B and the phase diagram 9

F The reputation-based model in a mixed population **11**
F.1 Three archetypal games 11
F.2 Dependence on continuous variation of game B and the phase diagram in the reputation-based model 12

G Phase transitions **12**

H Dependence on the mutation rate in a mixed population **14**

I Structured population **14**
I.1 Direct interaction model with three archetypal games 14
I.2 Dependence on the continuous variations of the structure of game B and mutation rates 16

J Codes **16**
J.1 Codes for the direct interaction Model 17
J.1.1 Simulations 17
J.1.2 Numerical solution of the replicator dynamics 20

J.2 Codes for the Reputation-based model 22
J.2.1 Simulations 22
J.2.2 Numerical solution of the replicator dynamics 26

A Overview of the models

In the manuscript we have considered two different scenarios, both in a mixed population, and in a structured population. In the following we explain the models separately, beginning with the mixed population case.

A.1 The mixed population

A.1.1 Model 1: The direct interaction model

In the first model, we consider a population of N individuals. At each time step, individuals are randomly paired to play the games. Each pair plays a prisoner's dilemma (PD) followed by a second game, which we call game B. The second game is a two-person, two-strategy game. The two strategies of the second game are called up (u) and down (d). Individuals decide upon their strategy in the second game based on their opponents' strategy in the first game. Thus, we can show a strategy by a triple $x_1x_2x_3$. Here, x_1 is the strategy of the individual in the first game and can be cooperation (C) or defection (D), x_2 is the strategy the individual plays in game B in case its opponent cooperates in the PD, and x_3 is the strategy the individual plays in game B in case its opponent defects in the PD. Obviously, x_2 and x_3 can be u or d. Individuals derive payoffs according to the payoff structure of the games, and reproduce with a probability proportional to their payoff. During reproduction, the whole population is updated, such that each individual in the next generation is offspring to an individual in the past generation with a probability proportional to its payoff. Offspring inherit the strategy of their parent. However, with probability ν, a mutation occurs. In the case of mutation, the offspring's strategy is changed to another randomly chosen strategy, other than the strategy of its parent.

A.1.2 Model 2: The reputation-based model.

In the second model, we consider a population of N individuals. At each time step, individuals play two games, a prisoner's dilemma and a second game, with a different payoff structure. The second game, which we call game B, is a two-person two-strategy game. We call the two strategies of the game B, down and up strategies. The individuals' strategy in game B is a function of the reputation of their opponent in the PD game. Thus, the strategy of an individual is determined by a triple $x_1x_2x_3$. Here, x_1 is the individual's strategy in the PD, x_2 is its strategy if the individual perceives its opponent to be a cooperator, and x_3 is the individual's strategy in case it perceives the reputation of its opponent as a defector. For reputation dynamics, we assume each individual perceives its opponent's reputation correctly with probability $1-\eta$ and perceives a wrong reputation with probability η. In this way, reputation is governed by a single parameter, which is the probability of error and can be considered a misinformation measure. Each individual plays its two games with randomly chosen individuals. That is, at each time step, individuals are first randomly paired to play the PD, after which the interactions end. Then, individuals are randomly paired again, this time to play game B. Individuals derive payoff according to the payoff structure of the games and reproduce with a probability proportional to their payoff. Offspring inherit the strategy of their parent, up to mutations. Mutations occur with probability ν. In case a mutation occurs, the offspring's strategy is changed to another randomly chosen strategy, other than the strategy of its parent.

A.2 Structured population

In the structured population case, we assume the individuals reside on a network and derive payoffs by playing the games with their neighbors. For the network structure, we consider a first nearest neighbor square lattice with von Neumann connectivity and periodic boundaries. For the evolutionary process, we consider an imitation dynamic. In this evolutionary process, each individual imitates the strategy of an individual in its extended neighborhood chosen with a probability proportional to their payoff. The extended neighborhood of an individual is defined as the focal individual together with its neighbors. Mutations can occur as well. We assume for each individual in a reproduction process, a mutation occurs with probability ν. In the case of a mutation, the individual's strategy is set randomly equal to a strategy other than its parent's strategy.

We simulate the model on a network for both the direct interaction model and the reputation-based model. In the direct interaction model, individuals play both their games with each neighbor consecutively. In the reputation-based model, individuals first play their first game (PD) with all their neighbors. Then they play their second game (game B), with all their neighbors. In addition, in the reputation-based model, individuals make an error in inferring their opponent's reputation with probability η. In case of a recognition error, an individual perceives a cooperator to be a defector and vice versa. We note that, apart from different samplings, for $\eta = 0$ the two models give rise to similar dynamics.

B Details of the Methods and derivation of replicator dynamics

B.1 Games and their payoff

We take game B to be a symmetric two-person, two-strategy game. This can be represented in normal form as in Table A. In each entry of this Table, the first letter shows the payoff to the row player, and the second letter stands for the payoff to the column player. In addition to continuous variations of the payoff structure of game B, we have used three archetypal games. The base payoff values used for the three archetypal games are presented in Table. B.

Prisoner's dilemma

	cooperation	defection
cooperation	R, R	S, T
defection	T, S	P, P

Game B

	down	up
down	R_B, R_B	S_B, T_B
up	T_B, S_B	P_B, P_B

Table A: Normal form representation of the games. The first letter in each block shows the row player's payoff, and the second letter is the column player's payoff.

Prisoner's dilemma

	R	S	T	P
Prisoner's Dilemma	3	0	5	1

Game B

	R_B	S_B	T_B	P_B
Snow Drift (SD)	3	1	5	0
Battle of the Sexes (BS)	0	3	5	0
Leader	2	3	5	1

Table B: Base payoff values.

B.2 Soft and hard strategies

We call the two strategies of game B, up and down. As mentioned before, for the class of the games considered here, that is, games with an asymmetric Nash equilibrium, it is possible to make a distinction between a soft and a hard strategy. We call a strategy soft, if in the Nash equilibrium it leads to a higher payoff for the opponent compared to the focal player. Similarly, we call a strategy a hard strategy, if in Nash equilibrium it leads to a higher payoff for the focal player compared to the opponent. For the three archetypal games, we take the strategy d to be soft and the strategy u to be the hard strategy. However, when studying continuous variations of game B, it can happen that strategy d, or u becomes a soft or hard strategy depending on the payoff values of game B.

B.3 The replicator dynamics

The model can be solved in terms of the replicator-mutator equation, which reads as follows:

$$\rho_x(t+1) = \sum_y \nu_{x,y}\rho_y(t)\frac{\pi_y}{\bar{\pi}}. \tag{A}$$

Here, ρ_x is the density of strategy x, π_y, is the expected payoff of strategy y, $\bar{\pi}$ is the mean payoff, and $\nu_{x,y}$ is the mutation rate from strategy y to the strategy x. This can be written as:

$$\nu_{y,x} = \begin{cases} 1-\nu & \textit{if} \quad y = x, \\ \nu/7 & \textit{if} \quad y \neq x. \end{cases} \tag{B}$$

The payoff of an strategy can be written as follows. First we define:

$$\begin{aligned}
&\rho_{C,u(C)} = \rho_{Cuu} + \rho_{Cud}, \quad \rho_{C,d(C)} = \rho_{Cdu} + \rho_{Cdd}, \\
&\rho_{C,u(D)} = \rho_{Cuu} + \rho_{Cdu}, \quad \rho_{C,d(D)} = \rho_{Cud} + \rho_{Cdd}, \\
&\rho_{D,u(C)} = \rho_{Duu} + \rho_{Dud}, \quad \rho_{D,d(C)} = \rho_{Ddu} + \rho_{Ddd}, \\
&\rho_{D,u(D)} = \rho_{Duu} + \rho_{Ddu}, \quad \rho_{D,d(D)} = \rho_{Dud} + \rho_{Ddd}.
\end{aligned} \tag{C}$$

Here, the first letter in the indices shows the strategy in the PD, and $s(C)$ ($s(D)$), is the strategy in game B against a cooperator (defector). That is, for example, $\rho_{C,u(C)}$ is the density of those individuals who cooperate in the PD and play the up strategy with cooperators. In addition, in the following, we use ρ_C and ρ_D for the total density of those who, respectively, cooperate or defect in the PD. That is:

$$\begin{aligned}
&\rho_C = \rho_{Cuu} + \rho_{Cud} + \rho_{Cdu} + \rho_{Cdd}, \\
&\rho_D = \rho_{Duu} + \rho_{Dud} + \rho_{Ddu} + \rho_{Ddd}.
\end{aligned} \tag{D}$$

Given these definitions, the payoffs of different strategies in the direct interaction model can be written as follows:

$$\begin{aligned}
\pi_{Cuu} &= \rho_C R + \rho_D S + \rho_{C,u(C)} P_B + \rho_{D,u(C)} P_B + \rho_{C,d(C)} T_B + \rho_{D,d(C)} T_B, \\
\pi_{Cud} &= \rho_C R + \rho_D S + \rho_{C,u(C)} P_B + \rho_{D,u(C)} S_B + \rho_{C,d(C)} T_B + \rho_{D,d(C)} R_B, \\
\pi_{Cdu} &= \rho_C R + \rho_D S + \rho_{C,u(C)} S_B + \rho_{D,u(C)} P_B + \rho_{C,d(C)} R_B + \rho_{D,d(C)} T_B, \\
\pi_{Cdd} &= \rho_C R + \rho_D S + \rho_{C,u(C)} S_B + \rho_{D,u(C)} S_B + \rho_{C,d(C)} R_B + \rho_{D,d(C)} R_B, \\
\pi_{Duu} &= \rho_C T + \rho_D P + \rho_{C,u(D)} P_B + \rho_{D,u(D)} P_B + \rho_{C,d(D)} T_B + \rho_{D,d(D)} T_B, \\
\pi_{Dud} &= \rho_C T + \rho_D P + \rho_{C,u(D)} S_B + \rho_{D,u(D)} S_B + \rho_{C,d(D)} R_B + \rho_{D,d(D)} R_B, \\
\pi_{Ddu} &= \rho_C T + \rho_D P + \rho_{C,u(D)} P_B + \rho_{D,u(D)} P_B + \rho_{C,d(D)} T_B + \rho_{D,d(D)} T_B, \\
\pi_{Ddd} &= \rho_C T + \rho_D P + \rho_{C,u(D)} S_B + \rho_{D,u(D)} S_B + \rho_{C,d(D)} R_B + \rho_{D,d(D)} R_B.
\end{aligned} \tag{E}$$

Here the first two terms in each expression are the payoffs from the PD, and the last four terms are the payoff from game B. The validity of these expressions can be checked by enumerating all the possible strategies that a focal individual can play against. For example, the third term in the expression for π_{Cuu} can be written by noting that a focal Cuu player, meets an individuals of type $C, u(C)$ with probability $\rho_{C,u(C)}$. In this interaction both the focal individual and the opponent play u, leading to a payoff of P_B for the focal individual.

In the reputation-based model, we have for the payoff of individuals from the PD:

$$\begin{aligned}
&\pi^A_{Cuu} = \rho_C R + \rho_D S, \quad \pi^A_{Cud} = \rho_C R + \rho_D S, \quad \pi^A_{Cdu} = \rho_C R + \rho_D S, \quad \pi^A_{Cdd} = \rho_C R + \rho_D S, \\
&\pi^A_{Duu} = \rho_C T + \rho_D P, \quad \pi^A_{Dud} = \rho_C T + \rho_D P, \quad \pi^A_{Ddu} = \rho_C T + \rho_D P, \quad \pi^A_{Ddd} = \rho_C T + \rho_D P.
\end{aligned} \tag{F}$$

The payoffs from game B can be written as:

$$\begin{aligned}
\pi^B_{Cuu} =& \rho_{Cuu} P_B + (1-\eta)\rho_{Cud} P_B + \eta\rho_{Cud} T_B + \rho_{Duu} P_B + (1-\eta)\rho_{Dud} P_B + \eta\rho_{Dud} T_B + \\
& \rho_{Cdd} T_B + (1-\eta)\rho_{Cdu} T_B + \eta\rho_{Cdu} P_B + \rho_{Ddd} T_B + (1-\eta)\rho_{Ddu} T_B + \eta\rho_{Ddu} P_B, \\
\pi^B_{Cud} =& (1-\eta)\big[\rho_{Cuu} P_B + (1-\eta)\rho_{Cud} P_B + \eta\rho_{Cud} T_B\big] + \eta\big[\rho_{Cuu} S_B + (1-\eta)\rho_{Cud} S_B + \eta\rho_{Cud} R_B\big] + \\
& (1-\eta)\big[\rho_{Duu} S_B + (1-\eta)\rho_{Dud} S_B + \eta\rho_{Dud} R_B\big] + \eta\big[\rho_{Duu} P_B + (1-\eta)\rho_{Dud} P_B + \eta\rho_{Dud} T_B\big] + \\
& (1-\eta)\big[\rho_{Cdd} T_B + (1-\eta)\rho_{Cdu} T_B + \eta\rho_{Cdu} P_B\big] + \eta\big[\rho_{Cdd} R_B + (1-\eta)\rho_{Cdu} R_B + \eta\rho_{Cdu} S_B\big] + \\
& (1-\eta)\big[\rho_{Ddd} R_B + (1-\eta)\rho_{Ddu} R_B + \eta\rho_{Ddu} S_B\big] + \eta\big[\rho_{Ddd} T_B + (1-\eta)\rho_{Ddu} T_B + \eta\rho_{Ddu} P_B\big], \\
\pi^B_{Cdu} =& (1-\eta)\big[\rho_{Cuu} S_B + (1-\eta)\rho_{Cud} S_B + \eta\rho_{Cud} R_B\big] + \eta\big[\rho_{Cuu} P_B + (1-\eta)\rho_{Cud} P_B + \eta\rho_{Cud} T_B\big] + \\
& (1-\eta)\big[\rho_{Duu} P_B + (1-\eta)\rho_{Dud} P_B + \eta\rho_{Dud} T_B\big] + \eta\big[\rho_{Duu} S_B + (1-\eta)\rho_{Dud} S_B + \eta\rho_{Dud} R_B\big] + \\
& (1-\eta)\big[\rho_{Cdd} R_B + (1-\eta)\rho_{Cdu} R_B + \eta\rho_{Cdu} S_B) + \eta\big[\rho_{Cdd} T_B + (1-\eta)\rho_{Cdu} T_B + \eta\rho_{Cdu} P_B\big] + \\
& (1-\eta)\big[\rho_{Ddd} T_B + (1-\eta)\rho_{Ddu} T_B + \eta\rho_{Ddu} P_B\big] + \eta\big[\rho_{Ddd} R_B + (1-\eta)\rho_{Ddu} R_B + \eta\rho_{Ddu} S_B\big], \\
\pi^B_{Cdd} =& \rho_{Cuu} S_B + (1-\eta)\rho_{Cud} S_B + \eta\rho_{Cud} R_B + \rho_{Duu} S_B + (1-\eta)\rho_{Dud} S_B + \eta\rho_{Dud} R_B + \\
& (\rho_{Cdd} R_B + (1-\eta)\rho_{Cdu} R_B + \eta\rho_{Cdu} S_B) + \rho_{Ddd} R_B + (1-\eta)\rho_{Ddu} R_B + \eta\rho_{Ddu} S_B, \\
\pi^B_{Duu} =& \rho_{Cuu} P_B + (1-\eta)\rho_{Cud} T_B + \eta\rho_{Cud} P_B + \rho_{Duu} P_B + (1-\eta)\rho_{Dud} T_B + \eta\rho_{Dud} P_B + \\
& \rho_{Cdd} T_B + (1-\eta)\rho_{Cdu} P_B + \eta\rho_{Cdu} T_B + \rho_{Ddd} T_B + (1-\eta)\rho_{Ddu} P_B + \eta\rho_{Ddu} T_B,
\end{aligned}$$

$$
\begin{aligned}
\pi^B_{Dud} =&(1-\eta)\left[\rho_{Cuu}P_B + (1-\eta)\rho_{Cud}T_B + \eta\rho_{Cud}P_B\right] + \eta\left[\rho_{Cuu}S_B + (1-\eta)\rho_{Cud}R_B + \eta\rho_{Cud}S_B\right]+\\
&(1-\eta)\left[\rho_{Duu}S_B + (1-\eta)\rho_{Dud}R_B + \eta\rho_{Dud}S_B\right] + \eta\left[\rho_{Duu}P_B + (1-\eta)\rho_{Dud}T_B + \eta\rho_{Dud}P_B\right]+\\
&(1-\eta)\left[\rho_{Cdd}T_B + (1-\eta)\rho_{Cdu}P_B + \eta\rho_{Cdu}T_B\right] + \eta\left[\rho_{Cdd}R_B + (1-\eta)\rho_{Cdu}S_B + \eta\rho_{Cdu}R_B\right]+\\
&(1-\eta)\left[\rho_{Ddd}R_B + (1-\eta)\rho_{Ddu}S_B + \eta\rho_{Ddu}R_B\right] + \eta\left[\rho_{Ddd}T_B + (1-\eta)\rho_{Ddu}P_B + \eta\rho_{Ddu}T_B\right],\\
\pi^B_{Ddu} =&(1-\eta)\left[\rho_{Cuu}S_B + (1-\eta)\rho_{Cud}R_B + \eta\rho_{Cud}S_B\right] + \eta\left[\rho_{Cuu}P_B + (1-\eta)\rho_{Cud}T_B + \eta\rho_{Cud}P_B\right]+\\
&(1-\eta)\left[\rho_{Duu}P_B + (1-\eta)\rho_{Dud}T_B + \eta\rho_{Dud}P_B\right] + \eta\left[\rho_{Duu}S_B + (1-\eta)\rho_{Dud}R_B + \eta\rho_{Dud}S_B\right]+\\
&(1-\eta)\left[\rho_{Cdd}R_B + (1-\eta)\rho_{Cdu}S_B + \eta\rho_{Cdu}R_B\right] + \eta\left[\rho_{Cdd}T_B + (1-\eta)\rho_{Cdu}P_B + \eta\rho_{Cdu}T_B\right]+\\
&(1-\eta)\left[\rho_{Ddd}T_B + (1-\eta)\rho_{Ddu}P_B + \eta\rho_{Ddu}T_B\right] + \eta\left[\rho_{Ddd}R_B + (1-\eta)\rho_{Ddu}S_B + \eta\rho_{Ddu}R_B\right],\\
\pi^B_{Ddd} =&\rho_{Cuu}S_B + (1-\eta)\rho_{Cud}R_B + \eta\rho_{Cud}S_B + \rho_{Duu}S_B + (1-\eta)\rho_{Cud}R_B + \eta\rho_{Cud}S_B+\\
&(\rho_{Cdd}R_B + (1-\eta)\rho_{Cdu}S_B + \eta\rho_{Cdu}R_B) + \rho_{Ddd}R_B + (1-\eta)\rho_{Ddu}S_B + \eta\rho_{Ddu}R_B. \qquad \text{(G)}
\end{aligned}
$$

These expressions can be written by considering a focal individual with a given strategy x. For the strategies which play differently with cooperators and defectors, that is Cud, Cdu, Dud, and Ddu, the brackets with the coefficient $1-\eta$ result from the case that the focal individual correctly infers the strategy of its opponent, and the brackets with the coefficient η result from the case that the focal individual makes an error in inferring the strategy of its opponent. The opponent infers the PD-strategy of the focal individual correctly with probability $1-\eta$ and makes an error with probability η. By considering the four possible cases that, the focal individual makes an error and the opponents makes a correct inference, the focal individual makes a correct inference and the opponent makes an error, both make an error, and both make a correct inference, and enumerating all the possible strategies for the opponent, it is possible to determine the payoff of the focal individual in each case. For example, a focal Cud receives payoff P_B in an interaction with a Cud opponent if both make a correct inference, and T_B if the focal individual makes a correct inference and the opponent makes an error. Each of these interactions take place with probability $\rho_{Cud}(1-\eta)^2$ for the former and $\rho_{Cud}(1-\eta)\eta$ for the latter. Enumerating all the possible strategies for the opponent, y, multiplying with the probability that the focal individual meets an opponent with strategy y, ρ_y, and multiplying the probabilities that each of the aforementioned four cases occurs, we arrive at these equations.

We note that, for the strategies which play in the same way with cooperators and defectors, that is Cuu, Cdd, Duu, and Ddd, the focal individual plays the same strategy irrespective of making an inference error or not. Consequently the payoff of the focal individual depends only on the perception error of the opponent. Enumerating all the possible strategies for the opponent we can write the corresponding equations for the payoffs in eq. G.

B.4 Calculation of the connected correlation functions

The connected correlation function is calculated by assigning $+1$ to cooperation in the PD and strategy down in game B, and -1 to defection in PD and strategy up in game B. In the simulations, we calculate the connected correlation function, $\langle s_A s_B\rangle_c = \langle s_A s_B\rangle - \langle s_A\rangle\langle s_B\rangle$, by calculating $s_A s_B$ for each individual and averaging over the population. For replicator dynamics, in the direct interaction model, this can be calculated using the following equations:

$$
\begin{aligned}
\langle s_A s_B\rangle =& -\rho_{Cuu} + \rho_{Cud}(-\rho_C + \rho_D) + \rho_{Cdu}(\rho_C - \rho_D) + \rho_{Cdd} + \rho_{Duu} + \rho_{Dud}(\rho_C - \rho_D)\\
&+ \rho_{Ddu}(-\rho_C + \rho_D) - \rho_{Ddd}, \qquad \text{(H)}
\end{aligned}
$$

and

$$\langle s_A\rangle\langle s_B\rangle = (\rho_C - \rho_D)(\rho_d - \rho_u). \qquad \text{(I)}$$

Eq. H can be derived by calculating the contribution of each strategy into $\langle s_A s_B \rangle$. For example, the strategy Cuu always cooperates in the PD and plays u in game$B$. This gives $s_A s_B = -1$. As an individual is of this type with probability ρ_{Cuu}, the contribution of this strategy to the correlation function is the first term in eq. H. Using a similar argument, it is easy to derive other terms in this equation.

In the reputation-based model, the connected correlation function can be calculated using the following equations:

$$\begin{aligned}\langle s_A s_B \rangle = &-\rho_{Cuu} + \rho_{Cud}\big[(1-\eta)(-\rho_C+\rho_D) - \eta(-\rho_C+\rho_D)\big] + \rho_{Cdu}\big[(1-\eta)(\rho_C-\rho_D) - \eta(\rho_C-\rho_D)\big] \\ &+ \rho_{Cdd} + \rho_{Duu} + \rho_{Dud}(\big[(1-\eta)(\rho_C-\rho_D) - \eta(\rho_C-\rho_D)\big] \\ &+ \rho_{Ddu}\big[(1-\eta)(-\rho_C+\rho_D) - \eta(-\rho_C+\rho_D)\big] - \rho_{Ddd}. \end{aligned} \tag{J}$$

$$\langle s_A \rangle \langle s_B \rangle = (\rho_C - \rho_D)(\rho_d - \rho_u). \tag{K}$$

Eq. J, can be derived by calculating the contribution of each strategy to the connected correlation function. For example, the strategy Cuu always cooperates and plays u. Thus, we have $s_A s_B = -1$. As this strategy occurs with probability ρ_{Cuu} in the population, its contribution to $\langle s_A s_B \rangle$ is equal to $-\rho_{Cuu}$. Similarly, strategy Cud cooperates in the PD. With probability ρ_C an individual with this strategy meets a cooperator. In this case, it plays u with probability $1-\eta$ and plays d with probability η. In the former case, $s_A s_B = -1$ and in the latter case $s_A s_B = +1$. With probability ρ_D an individual with this strategy meets a defector. In this case, it plays d with probability $1-\eta$ and plays u with probability η. In the former case, $s_A s_B = +1$ and in the latter case $s_A s_B = -1$. Averaging over the population gives the second term for the contribution of the strategy Cud to the connected correlation function. The contribution of other strategies can be calculated in a similar way.

C Nash equilibria when game B is the Battle of the Sexes and the Leader game

The payoff values of the two-stage game when game $B$is the Battle of the Sexes (top) and the Leader game (bottom) are presented in Table. C. The Nash equilibria are denoted by green cells. Similarly to the case that the game B is a Snow Drift game, studied in the main text, the two-stage game has two classes of Nash equilibria, cooperative and defective equilibria. Defective equilibria are composed of four equilibria in which mutual defection in the PD and heterogeneous (u,d) strategy pair in the game B is played. Both these strategy profiles are the Nash equilibrium of the composing games. On the other hand, cooperative equilibria are composed of two Nash equilibria: (Cuu, Ddu) and (Cdu, Ddu). In both these equilibria, the cooperator plays the hard strategy with the defector, and the defector plays the soft strategy with the cooperator.

D Supplementary Videos

The Supplementary Videos (SV) show the time evolution of the model in a structured population. In the videos, a population of 160000 individuals resides on a first nearest neighbor square lattice with von Neumann connectivity. The mutation rate is set equal to $\nu = 10^{-4}$. The payoff values are chosen according to Table. B. Different strategies are color coded as indicated in the video. In SV.1 game B is the Snow Drift game, in SV. 2, it is the Battle of the Sexes, and in SV. 3, game B is the Leader game. The initial condition is chosen as a defection favoring one. That is, one in which all the individuals are of type Dud.

Game B: Battle of the Sexes

	Cuu	Cud	Cdu	Cdd	Duu	Dud	Ddu	Ddd
Cuu	3,3	3, 3	8,6	8,6	0,5	0,5	5,8	5,8
Cud	3,3	3,3	8,6	8,6	3,10	3,10	0,5	0,5
Cdu	6,8	6,8	3,3	3,3	0,5	0,5	5,8	5,8
Cdd	6,8	6,8	3,3	3,3	3,10	3,10	0,5	0,5
Duu	5,0	10,3	5,0	10,3	1,1	6,4	1,1	6 , 4
Dud	5,0	10,3	5,0	10,3	4,6	1,1	4,6	1,1
Ddu	8,5	5,0	8,5	5,0	1,1	6,4	1,1	6,4
Ddd	8,5	5,0	8,5	5,0	4,6	1,1	4,6	1,1

Game B: Leader game

	Cuu	Cud	Cdu	Cdd	Duu	Dud	Ddu	Ddd
Cuu	4,4	4,4	8,6	8,6	1,6	1,6	5,8	5,8
Cud	4,4	4,4	8,6	8,6	3,10	3,10	2,7	2,7
Cdu	6,8	6,8	5,5	5,5	1,6	1,6	5,8	5,8
Cdd	6,8	6,8	5,5	5,5	3,10	3,10	2,7	2,7
Duu	6,1	10,3	6,1	10,3	2,2	6,4	2,2	6,4
Dud	6,1	10,3	6,1	10,3	4,6	3,3	4,6	3,3
Ddu	8,5	7,2	8,5	7,2	2,2	6,4	2,2	6,4
Ddd	8,5	7,2	8,5	7,2	4,6	3,3	4,6	3,3

Table C: Payoff values of the two-stage game when game B is the Battle of the Sexes (top) and Leader game (bottom). Cooperative Nash equilibria are denoted by green cells and defective Nash equilibria are denoted by red cells.

E Analysis of direct interaction model in a mixed population

This section analyzes the direct interaction model, where individuals play both a PD and a second game with the same opponent. We begin by separately considering the three archetypal games: SD, BS, and the Leader game. Then we proceed to study the phase diagram of the model as a function of a continuous deformation of the structure of game B.

E.1 Three archetypal game

We begin by looking at the time evolution of the system, by plotting the density of different strategies as a function of time, in the cases that the game B is the BS and the Leader game in, respectively, Figs. A and B. The same behavior observed in the main text, in the case of the SD game, is observed here. Starting from a random initial condition, the dynamics rapidly settle in a state where the strategies' density resembles those in the defective fixed point. In this case, the density of strategies in both the PD and game B are close to their Nash equilibrium value. This sets the stage for the second step of the evolution where those strategies which play soft with cooperators slowly accumulate and increase in number. This is due to the fact that because of the low density of cooperators, such strategies do not impose a high cost on their bearer. Accumulation of these cooperation favoring strategies leads to a situation where cooperators' benefit in the second game can compensate for the cost of cooperation in the first round. At this point, the dynamics show a rapid transition to a state where cooperators are found in the system in large densities. At the stationary state, the population fixates in a moral system according to which defectors always play the soft strategy with cooperators, and cooperators play hard with defectors. The majority of cooperators belong to one of the two types. One type, Cdu, plays soft with fellow cooperators, while the other type, Cuu, always plays hard, with

cooperators and defectors alike. Similarly, defectors belong to two types. One type, Ddd, plays soft with both cooperators and defectors, and the other one, Ddu, plays hard with defectors but soft with cooperators. This shows cooperators are more likely to play a hard strategy in the second round than the defectors are. This, in turn, leads to an anti-correlation between the strategies of the individuals in the two rounds.

The densities of different strategies in the stationary state as a function of the temptation, T, are plotted in Figs. C, D, and E, in the cases where the game B is, respectively, SD, BS, and the Leader game. Here the results of the replicator dynamics is shown by lines. The solid blue line shows the equilibrium fixed point: This is the attractor of the dynamics starting from an unbiased initial condition in which the frequencies of all the strategies are equal. The dashed red line shows the non-equilibrium fixed point: This stationary state can occur starting from certain initial conditions. While in all the cases, the dynamic is bistable, in equilibrium, cooperation evolves. The markers show the results of simulations in a population of size $N = 10000$. Here, a sample of $R = 80$ simulations is used. Simulations are run for $T = 20000$ time steps, and averages over the last 1000 time steps are used. In each simulation, the dynamics settle in one of the two fixed points. The markers show the average over the set of runs that the dynamics settle in the given fixed point, and the marker sizes are proportional to the number of times that the dynamics settle in that fixed point. The error bars show the in-sample standard deviations. That is the standard deviation in the subset of simulations that settle in a given stationary state. Simulation results show, in a finite population, starting from a random initial condition, the system goes to the equilibrium fixed point with a high probability. However, in some cases, it can happen that the dynamics settle in the defective fixed point. This is the case only when game B is the BS game. In addition, in all the cases, in the cooperative fixed point, defectors always play soft with cooperators, and cooperators play hard with defectors. On the other hand, cooperators and defectors play a combination of soft and hard strategy among themselves.

The payoff of cooperators and defectors in the first game (game A, which is the prisoner's dilemma), and the second game, game B, is plotted in Fig. F. Here, from top to bottom, game B is an SD, BS, and a Leader game. As expected, the payoff of cooperators is smaller than that of the defectors in the PD. This loss of payoff is however compensated by the larger payoff that cooperators achieve in the second game. As mentioned before, this is due to the fact that due to the emergence of a set of moral rules, cooperators are allowed to play hard with defectors. This can be seen more clearly to be the case in Fig. G, where the density of strategies that play hard with cooperators, $u(C)$, soft with cooperators $d(C)$, hard with defectors $u(D)$, and soft with defectors, $d(D)$, are plotted, respectively, in panels (a) to (d). Here, from top to bottom, game B is an SD, BS, and a Leader game. As can be seen, in all the cases, cooperators are more likely to be played soft with, than the defectors are. In addition, the likelihood that a cooperator receives a soft encounter increases with increasing the strength of the social dilemma, i.e., by increasing T. This shows, interestingly, the more strong a dilemma, and the higher the cost of cooperation, a stronger set of cooperation supporting moral rules emerge.

E.2 Dependence on continuous variation of game B and the phase diagram

An interesting question is how the model behaves with respect to continuous deformation of the payoff structure of game B? To see this, in Fig. H and Fig. I, we color plot the densities of different strategies, in the $S_B - T_B$ plane. Here, we have fixed, $R = 3$, $S = 0$, $P = 1$, and $T = 5$, for the PD, and for game B, we have fixed $R_B = 3$, and $P_B = 1$. In Fig. H the result of the replicator dynamics is shown, and in Fig. I, the result of simulations in a population of size 1000 is used. For the simulations, a sample of 128 simulations is used. The simulations are run for 10000 time steps, and an average over the last 1000 time steps is taken. Here, the initial condition is a an unbiased initial condition,

in which the initial density of all the strategies equal. For the replicator dynamics this amounts to setting, $\rho_x = 1/8$, where x can be any of the 8 possible strategies. For the simulations, this is assured by random assignment of the strategies of the individuals.

In all the panels in Figs. H and I, the phase diagram of the model is superimposed as well. For $T_B < 3$, game B becomes a coordination game: In this case the Nash equilibrium is a symmetric strategy pair in which both players play d. On the other hand, for $T_B > 3$ and S_B smaller than $P_B = 1$, game B becomes the prisoner's dilemma, with a symmetric mutual defection Nash equilibrium. On the other hand, for $T_B > 3$ and $S_B > P_B = 1$, game B has an asymmetric Nash equilibrium, and can give rise to cooperation in the PD. However, for too small values of S_B and T_B in this region, the dynamics remain mono-stable, with a defective fixed point which supports a low level of cooperation. For larger values of T_B andS_B, the dynamics become bistable: Here a cooperative fixed point in which a high level of cooperation evolves becomes possible as well. The boundary of bistability, above which the system becomes bistable is plotted by red and green lines. The two branches of the boundary of bi-stability meet at a critical point. At this point, the transition between the defective and cooperative fixed points become a continuous transition. We note that, above the critical point, there is a mono-stable region, where starting from any initial condition, a considerable level of cooperation evolves. The results of the replicator dynamics show in an infinite size system, starting from an unbiased initial condition, above a phase transition line the system settles in the cooperative fixed point. Comparison with the results of simulations in a population of size 1000, shows, finite size effects strongly favor cooperation: In this case, starting from an unbiased initial condition, the dynamics settle in the cooperative fixed point in the whole bistable region with a high probability.

We note that, above the green line, the strategy d entails a lower payoff and can be seen as the soft strategy. Consequently, strategies which play down with cooperators, Cdu, Ddd, and Ddu, as well as cooperators who play up with others, Cuu, are found in the population in high density. This can be seen to be the case in Fig. H (replicator dynamics) and Fig. I (simulations), where the density of different strategies is plotted. Here, cooperators dominate in two types. Those who play the hard strategy with both cooperators and defectors (Cuu), and those who play soft with fellow cooperators, but hard with defectors (Cdu). On the other hand, the two dominated types of defectors are Ddd, who always play soft, and Ddu, who play soft with cooperators but hard with defectors. This shows that, as a result of evolution, a set of moral rules emerges according to which defectors always play soft with defectors and cooperators always play hard with defectors. In addition, cooperators are more likely to play a hard strategy in game B compared to defectors. These result in a higher payoff for cooperators in game B, which compensates for the cost of cooperation and leads to the evolution and maintenance of cooperation.

On the other hand, above the red line, we have $S_B > T_B$, which implies the strategy up entails a lower payoff and can be considered as the soft strategy. Consequently, in this case, strategies which play up with cooperators are found in high density. As can be seen in Fig. H (replicator dynamics) and Fig. I (simulations), these include strategies Cdu, Duu and Ddu. In addition, the cooperator type, Cdd, who plays hard with both cooperators and defector is found in the population as well. As in this case, the strategy d is the hard strategy and the strategy u is the soft strategy, the same observations as in the previous case are at work here. Namely, cooperators always play hard with defectors, and defectors play soft with cooperators. On the other hand, both cooperators and defectors play a combination of soft and hard strategy among themselves.

F The reputation-based model in a mixed population

F.1 Three archetypal games

The density of different strategies, in the cases where game B is one of the three archetypal two-person, two-strategy games, is plotted in Fig. J, Fig. K, and Fig. L, in the cases where, respectively, game B is the, SD, BS, and the leader game. Here, the solid blue line shows the equilibrium fixed point. This is the final state of the dynamics starting from an unbiased initial condition in which all the strategies are equal. The dashed red line shows the non-equilibrium fixed point. This is a stationary state of the dynamics starting from certain initial conditions. The results of a simulation in a population of size 20000 are shown with markers. Here, a sample of 80 simulations, starting from a random initial condition in which the strategies of the individuals are randomly assigned is used. In each simulation, the dynamics settle in one of the fixed points. The size of the markers is proportional to the number of times that the dynamics settle in the given fixed point. The markers and their error bars show, respectively, the average and the standard deviation in the sample of simulations in which the given stationary state occurs.

For $\eta = 0.5$, individuals make an error in inferring the PD strategy of their opponent, exactly half of the times. Consequently, there is no net information in their inference. As the probability of error, η decreases beyond a threshold, the dynamics become bistable: In addition to the defective fixed point, a cooperative fixed point emerges. In the cooperative fixed point the strategies Cuu, Cdu, Ddd, and Ddu dominate the population. As in the case of all the three archetypal games the strategy d is the soft strategy and the strategy u is the hard strategy. This means that cooperators play hard when they perceive their opponent to be a defector, and defectors play soft when they perceive their opponent to be a cooperator. As η further decreases, a phase transition occurs below which, starting from an unbiased initial condition, the dynamics settle in the cooperative fixed point.

We note that, the situation is similar as η increases beyond 0.5: above a boundary of bi-stability the dynamics become bistable, and a cooperative fixed point emerges. The reason is that, with η larger than 0.5, the individuals' inference contains net information about the strategy of their opponent. This information can be used by the individuals to increase their payoff. However, in this case, as individuals are more likely to make an error in inferring the strategy of their opponent than making a correct inference, the dominant strategies result from those dominated in low-η cooperative fixed point by an exchange of the B-game strategy against cooperators and defectors. Thus, the dominant strategies in this case are Cuu, Cdu, Ddd, and Dud. With this transformation, cooperators always play soft if they perceive their opponent to be a defector, and play hard if they perceive their opponent to be a cooperator. Combined with the fact that in this case individuals make an error in inference most of the times, this ensures that cooperators often (that is when they make an inference error) play hard with defectors, and defectors often play soft with cooperators.

The results of the simulations show similar fixed points exist in finite populations. However, finite size effects favor cooperation. This can be observed by noting that, in the bistable region, starting from a random initial condition, the dynamics in a finite population settle into the cooperative fixed point with a high probability, even when in an infinite population the equilibrium fixed point, is the defective fixed point.

The payoff of different strategies are plotted in Fig. M. Here, from panel (a) to (d), respectively, the payoff of cooperators in the first game, π_C^A, the payoff of defectors in the first game, π_D^A, the payoff of cooperators in the second game π_C^B, and the payoff of defectors in the second game π_D^B is plotted. In all the panels, from top to bottom, game B is, respectively, SD, BS, and the Leader game. As expected, in all the cases, in the prisoner's dilemma game the payoff of cooperators is smaller than that of the defectors. However, in the cooperative fixed point, this loss of payoff is compensated by a

larger payoff that cooperators receive from game B. On the other hand, in the defective fixed point, the payoff difference of cooperators and defectors from their second game is much smaller. However, even in this case, the payoff of cooperators can be larger than that of the defectors. This is the case for both the SD and the BS games.

F.2 Dependence on continuous variation of game B and the phase diagram in the reputation-based model

To see how the reputation-based model behaves with respect to the continuous variations of the payoff structure of game B, in Figs. N and O, we plot the density of different strategies in the reputation-based model, in the $S_B - T_B$ plane. Here the phase diagram of the model is super imposed as well. Fig. N shows the results of the replicator dynamics, and Fig. O shows the results of simulations in a populations of 1000 individuals. For the results of simulations, an average over a sample of 128 simulations is used. Simulations are run for 10000 time steps, and an average over the last 1000 steps is taken. Here, we have fixed, $R = 3$, $S = 0$, $P = 1$, and $T = 5$, for the PD, and for game B, we have fixed $R_B = 3$, and $P_B = 1$. The initial condition is an unbiased initial condition, in which the initial densities of all the strategies are equal. For the replicator dynamics this amounts to setting, $\rho_x = 1/8$, where x can be any of the 8 possible strategies. For the simulations, this is assured by random assignment of the individuals' strategies.

Here, the situation is similar to the direct interaction model. For $T_B < 3$, game B has a symmetric Nash equilibrium in which both players play d. On the other hand, for $T_B > 3$ and S_B smaller than $P_B = 1$, game B becomes the prisoner's dilemma, with a symmetric mutual defection (up) Nash equilibrium. On the other hand, for $T_B > 3$ and $S_B > P_B = 1$, game B has an asymmetric Nash equilibrium, and can give rise to cooperation in the PD. However, for too small values of S_B and T_B in this region, the dynamics remain mono-stable, with a defective fixed point which supports a low level of cooperation. For larger values of T_B and S_B, the dynamics become bistable: Here a cooperative fixed point in which a high level of cooperation evolves becomes possible as well. The boundary of bistability, above which the system becomes bistable is plotted by red and green lines. The two branches of the boundary of bistability meet at a critical point. At this point, the transition between the defective and cooperative fixed points become a continuous transition. As was the case in the direct interaction model, above the critical point, there is a mono-stable region, where starting from any initial condition, a considerable level of cooperation evolves.

The results of the replicator dynamics show in an infinite size system, starting from an unbiased initial condition, above a phase transition line the system settles in the cooperative fixed point. Comparison with the results of simulations in a population of size 1000, shows, finite size effects strongly favor cooperation: In this case, starting from an unbiased initial condition, the dynamics settle in the cooperative fixed point with a high probability in the whole bistable region.

G Phase transitions

As we have seen, in the $S_B - T_B$ plane, both models are mono-stable for small T_B and S_B. As either of these parameters increases, a cooperative fixed point becomes stable as well, and the dynamics become bistable. The boundary of bistability has two branches. Above one branch for large T_B (the green markers in Figs. N and H)), the soft strategy is the strategy down. Here, the strategies Cuu, Cdu, Ddu, and Ddd dominate. With the condition that the soft strategy is the strategy down, these are the strategies according to which cooperators play hard with defectors, and defectors play soft with cooperators. Above the other branch (the red markers in Figs. N and H)), S_B takes a large value. Here, the soft strategy is the strategy u. Consequently, the dominant strategies in the cooperative

fixed point are Cdd, Cud, Dud, and Duu. With the condition that u is the soft strategy, these strategy profiles are the ones according to which cooperators play hard with defectors, and defectors play soft with cooperators.

The two branches of the boundary of bistability meet at a certain critical point. In this point, the transition between the two fixed points becomes a continuous transition. To study this in more depth, in Figs. P(a) and P(d), we plot the density of cooperators, $\rho_C = \rho_{Cdd} + \rho_{Cdu} + \rho_{Cud} + \rho_{Cuu}$ in the fixed point of the dynamics as a function of T_B, for three different values of S_B, chosen close to the critical point. Here, the replicator dynamics is solved for two different initial conditions. Circles show the results for a cooperation favoring initial condition in which all the individuals belong to the type Cuu, and squares show a defection favoring initial condition in which all the individual are of type Cud. For S_B smaller than the critical value S_B^*, the system is bistable (blue solid line). Here, as T_B increases, a discontinuous transition from the defective fixed point to the cooperative fixed point occurs. The discontinuity of this transition is witnessed by the existence of a bistable region. On the other hand, for S_B larger than S_B^*, as T_B increases, there is a cross-over from the defective fixed point to the cooperative fixed point without passing any phase transition. In between these two extremes, the transition between the defective and cooperative phases becomes a continuous phase transition.

As it is well known, physical systems show a set of scaling relations close to a critical point. We proceed to confirm that such critical scaling relations indeed exists in our model. To do this, we use the density of cooperators in the stationary state as the order parameter of our system. The reduced order parameter is defined as the absolute value of the difference between the order parameter and its value at the critical point, $|\rho_C - \rho_C^*|$. Similarly, using T_B as the control parameter, we can define the reduced control parameter as the absolute value of the difference between the value of the control parameter from its critical value, $|T_B - T_B^*|$. Close to the phase transition, we expect to observe a power-law relation between these two variable:

$$|\rho_C - \rho_C^*| \propto |T_B - T_B^*|^{\alpha_\pm} \tag{L}$$

Here, α_+ and α_- are the critical exponents, respectively, above and below the phase transition. In principle, such a scaling relation may hold above and below the transition, with different exponents. By above (below) the transition, here we mean T_B larger (smaller) than the critical value T_B^*. Taking the logarithm of the two sides of proportionality in eq. L, it is easy to see that (L) implies the logarithm of the reduced order parameter as a function of the reduced control parameter should obey a linear relation with exponent $\alpha_\pm$. Thus, if the scaling holds, $|\rho_C - \rho_C^*|$ as a function of $|T_B - T_B^*|$, if plotted in a double logarithmic scale, should obey a linear relation, with the slope of the linear fit being equal to the critical exponent α_+ above, and α_- below the phase transition.

To see such a scaling relation indeed holds in the system, we plot the reduced order parameter as a function of the reduced control parameter, in Figs. P(b) and P(c) for the direct interaction model, and in Figs. P(e) and P(f), for the reputation-based model, in a double logarithmic plot. Here, by numerically solving the replicator dynamics we determine the critical point to be equal to $S_B^* = 3.995421 \pm 10^{-6}$ and $T_B^* = 4.4321683275147 \pm 10^{-12}$, for the direct interaction model, and $S_B^* = 4.19480358 \pm 10^{-8}$ and $T_B^* = 4.78261320545 \pm 10^{-11}$ for the reputation-based model. Figs. P(b) and P(e) show the scaling relation above the critical point, and Figs. P(c) and P(f) show the scaling relations below the critical point. As it can be seen, in both models, a scaling relation holds close to the critical point both above and below the phase transition, with a critical exponent which appears to be equal to 1/3 in all the cases. In general, it is expected that the values of the critical exponents to be universal and do not depend on the details of the statistical physical models. The fact that the exponents are the same for both direct interaction and the reputation-based model is in keeping with this universality principle.

To summarize, our results show that the system possesses a critical point in the $S_B - T_B$ plane. Below the critical point, the cooperative and defective strategies are symmetric, and are treated equally in game B. This leads to a defective state where the evolution of cooperation is prevented due to the cost of cooperation. Above the critical point however, the symmetry between cooperative and defective strategies break and a set of rules according to which cooperators are treated softly in game B emerges. Consequently, above the critical point, those strategies which are consistent with these behavioral rule dominate in the population.

H Dependence on the mutation rate in a mixed population

To study the dependence of our results on the mutation rate, we present the cooperation level in the $S_B - T_B$ plane in Figs. Q. In Q(a) and Q(c) the direct interaction model for, respectively, $\nu = 0.005$ and $\nu = 0.0005$ is considered. In Figs. Q(b) and Q(d) the reputation based model for respectively, $\nu = 0.005$ and $\nu = 0.0005$ is considered. Here, the replicator dynamics is solved starting from an unbiased initial condition in which the density of all the strategies are equal. We have set $R = 3$, $S = 0$, $P = 1$, $T = 5$, $R_B = 3$, and $P_B = 1$. In Figs. Q(b) and Q(d) $q = 0.1$. We have also plotted the boundaries of bistability. Below this boundary the dynamic is mono-stable, settling into a defective fixed point with a low level of cooperation. Above the boundary, a cooperative fixed point becomes stable and the dynamics become bistable. The two branches of the boundary meet at a critical point, where the transition becomes continuous.

Comparison between different mutation rate show that, smaller mutation rate favor cooperation. In both the direct interaction and the reputation-based model, for smaller mutation rate the boundaries of bistability above which the cooperative fixed point becomes stable shift to smaller values of T_B. In addition, starting from an unbiased initial condition, the system settles in the cooperative fixed point for a broader range of parameter values for smaller mutation rates.

I Structured population

I.1 Direct interaction model with three archetypal games

To study the direct interaction model, we perform simulations on a population of 20000 individuals residing on a 200 × 200 first nearest neighbor square lattice with von Neumann connectivity and periodic boundaries. The time average density of cooperators in the PD and the time average density of soft strategies in the game B as a function of temptation, for the cases that the game B is one of the three archetypal games is presented in, respectively, Figs. R(a) and R(b). Cooperation in the PD evolves for all the cases. Furthermore, cooperative behavior in game B reaches levels higher than the Nash equilibrium of the corresponding games in all the cases. The normalized payoff difference of cooperators and defectors in game B, $\Delta\pi = (\pi_C^B - \pi_D^B)/(\pi_B + \pi_D^B)$, is plotted in Fig. R(c). Where, it can be seen that cooperators reach a higher payoff in game B. This compensates for the cooperators' loss of payoff in the PD and leads to the evolution of cooperation. Finally, in Fig. R(d), we plot the connected correlation between the strategies of the individuals in game A and B. While this is negative when game B is the Leader or BS, it can be positive for the SD game. The reason is, contrary to what is the case in a mixed population, as explained below, for some parameter values, strategies of type Duu and Dud can survive in a structured population. These strategies contribute to the positivity of the correlation function.

A close examination of cooperation level for different structures of game B shows that When game B is the BS or the Leader game, cooperation decreases with increasing the temptation. This is due to the fact that by increasing the temptation the cost of cooperative behavior increases, and cooperators

experience a stronger disadvantage in the PD. This decreases the overall payoff of cooperators and leads to a decline in their density with increasing the temptation. the decline in the density of cooperators in turn leads to a decline in the density of cooperative behavior in game B as well. This is so because with fewer cooperators in the population, the diversity and complexity of interactions decreases, and individuals become more likely to interact with fellow defectors. This increases the simplicity of interactions, and thus, cooperative behavior in game B becomes closer to its Nash equilibrium value.

However, the situation is different for the case that game B is the Snow Drift game. In this case, by increasing the temptation, the density of cooperators decrease at first, and it starts to increase for larger values of T. This in turn increases the level of cooperative behavior in the Snow Drift game. Why the dependence of cooperation in temptation for the SD is different from that observed in the BS and the leader games? The clue to the answer of this question lies in the difference in the value of S_B in these cases. For both BS and Leader games, S_B is larger compared to the SD game. This means, when a down player encounters an up player, it experiences a stronger disadvantage in the SD game compared to the BS and the Leader games. In other words, the cost of playing soft in the SD can be larger than that in the BS and the Leader games. Consequently, Ddu individuals who defer to cooperators, and are often found in the cooperators' vicinity, reach a lower payoff from game B and are more dependent on their payoff from the PD (as in the PD by taking advantage from neighboring cooperators, they can compensate for their loss of payoff in game B due to deferring to cooperators) when game B is the SD game. For small T their payoff from the PD is small. Consequently they perform poor in competition with the domains of Duu and Dud. This decreases the territorial domain for cooperators and leads to a decline in cooperation level for small T. As T increases Ddu reaches a higher payoff (due to its larger payoff from the PD). This strengthens the coalition of Ddu and cooperators (Cuu and Cud), and consequently, they win in the territorial competition and invade the coexisting domains of Duu and Ddu. This increases cooperation level for larger T. However, as T becomes too large, the loss of payoff of cooperators increases, and this in turn affects cooperation level adversely. Consequently, cooperation level deceases for too large values of T. Thus, the analysis suggest when game B is the SD, an optimal value of temptation exist which optimizes cooperation.

The density of different strategies for the cases that the game B is one of the three archetypal games is potted in Fig. S. The two dominating cooperator types are Cuu and Cdu. As shown in the main text, these types survive by forming a coalition with the moral defectors, Ddu. When game B is BS or Leader, Ddu and Ddd are found in the highest density compared to other defector types. The density of anti-moral defectors, Duu and Dud, that is those who play hard with cooperators increases with increasing the temptation. This is due to the fact that for high values of the temptation, the loss of payoff of cooperators in the PD increases, such that the gain in payoff due to the existence of moral norms can not compensate for this loss of payoff. This decreases the density of cooperators. This deteriorate the ability of coexisting cooperators and moral defectors (Ddu type) to invade coexisting Duu and Dud domains. And consequently, domains of coexisting Duu and Dud grow in the system.

As described before, the situation is different when game B is the Snow Drift game. In this case, for low values of temptation, due to the lower payoff of Ddu type in the PD, coalition of cooperators and Ddu type performs poor in territorial competition with the coexisting domains of Duu and Dud. This causes the density of the latter two types to be higher for smaller values of temptation. for larger values of temptation, moral domains (coexisting cooperators and Ddu type) perform better in the territorial competition with anti-moral domains (coexisting Duu and Dud types). This leads to a decline in the density of anti-moral defectors by increasing the temptation.

Finally, to see how the strength of the social dilemma affects the evolution of moral norms, in Fig. T(a) to T(d), we plot the density of individuals who, respectively, play hard with cooperators, $u(C)$, those who play soft with cooperators, $d(C)$, those who play hard with defectors, $u(D)$, and

those who play soft with defectors $d(D)$. The density of individuals who play soft with cooperators is always larger than those who play soft with defectors. Similarly, the density of those who play hard with defectors is always larger than those who play hard with cooperators. This, consistently shows a set of cooperation supporting norms have evolved in the system. Furthermore, the density of those who play hard with cooperators decreases with increasing the temptation, while the density of those who play soft with cooperators increases with increasing the temptation. This shows, the stronger the social dilemma, the more likely that cooperators receive a favorable strategic response in game B. In other words, the cooperation supporting norms become stronger when the cost of cooperation is higher. This, in turn, by allowing cooperators to reach a higher payoff from the game B, partly compensates for the higher cost of cooperation that cooperators pay in a stronger social dilemma, and helps the evolution of cooperation when cooperation is more costly. However, the higher cost that cooperators pay in the PD may not be compensated by the stronger moral norms, and thus, cooperation can decline for higher values of temptation (as it is the case when game B is the Leader or the BS game).

I.2 Dependence on the continuous variations of the structure of game B and mutation rates

To see how the model behaves under continuous variations of the structure of the game B, we set $R = 3$, $S = 0$, $P = 1$, $T = 5$, $R_B = 3$, and $P_B = 1$, and plot the time average cooperation level in the prisoner's dilemma in the $S_B - T_B$ plane in Fig. U. In Fig. U(a) the direct interaction model is considered and in Fig. U(b) the reputation-based model is considered. In both cases we have set $\nu = 0.005$ and the simulations are performed in a population of $N = 40000$ individuals residing on a 200×200 square lattice with periodic boundaries and von Neumann connectivity. In Fig. U(b) $q = 0.1$.

Similarly to what was the case in a mixed population, in a structured population cooperation does not evolve when the game B has a symmetric Nash equilibrium. That is when $S_B < 1$ or $T_B < 3$. On the other hand, for $T_B > 3$ and $S_B > 1$, game B has an asymmetric Nash equilibrium. This satisfies the necessary condition for the evolution of cooperation. Cooperation evolves in practice, however, for large enough values of T_B or S_B.

To study the effect of mutation rate, we perform simulations with the same parameter values, but change the mutation rate to $\nu = 0.0005$. The result are presented in Fig. U(c) for the direct interaction model, and Fig. U(d) for the reputation-based model. As can be seen, smaller mutation rate can have a beneficial effect for the evolution of cooperation. This is specially the case for large values of T_B or S_B, such that game B is strongly asymmetric. In these cases, smaller mutation rate can lead to the evolution of a higher level of cooperation and for a broader range of parameter values, compared to larger mutation rates.

J Codes

In this section, the Matlab codes used for the case of a mixed population are presented. Four functions are presented below. In Sec. J.1.1, a function used for simulations in the direct interaction model is presented. In Sec. J.1.2, a function used for the numerical solution of the replicator dynamics in the direct interaction model is presented. In Sec. J.2.1, a function used for simulations in the reputation-based model is presented. Finally, in Sec. J.2.2, a function used for the numerical solution of the replicator dynamics in the reputation-based model is presented.

J.1 Codes for the direct interaction Model

J.1.1 Simulations

% This function is used for the simulations of the direct interaction model.
Output:
rho Cuu to rho Ddd are the density of different strategies (the same notation as in the main text is used). PiCA and piDA are the payoff of ,respectively, cooperators and defectors from the prisoner's dilemma, pidB and piuB are the payoffs of, respectively, down and up strategies from game B. mp is the average payoff, rhod is the density of the strategy down, and cor is the correlations between the strategies in the prisoner's dilemma and game B.
Input:
T is the duration of simulation, Ti is the initial time of recording the simulation results, N is the population size, nu1 is the mutation rate. Reward, Temptation, Punish, and Succer and the payoffs of the prisoner's dilemma. RewardB, TemptationB, PunishB, and SuccerB are the payoffs of game B, base is a base payoff, and caltime is the interval between recording the results

```
function [ rhoCuu , rhoCud , rhoCdu , rhoCdd , rhoDuu, rhoDud , rhoDdu , rhoDdd , piCA , piDA , pidB , piuB , mp , rhod , cor ] = fDirectInteraction( T , Ti , N , nu1 , Reward , Temptation , Punish , Succer , RewardB , TemptationB , PunishB , SuccerB , base , caltime)
%initial strategies in the prisoner's dilemma
strategyPD=randi(2,N,1);
%initial strategies in game B
strategyHD=randi(2,N,2);
sHD=zeros(N,1);
% payoff matrix of the prisoner's dilemma
WPD=[Reward,Succer;Temptation,Punish];
%payofff matrix of game B
WHD=[RewardB,SuccerB;TemptationB,PunishB];
payoff1=0*ones(N,1);
payoff2=0*ones(N,1);
tr=0;
for t=1:T;
    popvec=1:N;
    while  isempty(popvec);
        %random pairing of two individuals
        Len=length(popvec);
        a1=randi(Len);
        ind1=popvec(a1);
        popvec(a1)=[];
        a2=randi(Len-1);
        ind2=popvec(a2);
        popvec(a2)=[];
        %payoff from the prisoner's dilemma
        payoff1(ind1)=payoff1(ind1)+WPD(strategyPD(ind1),strategyPD(ind2));
        payoff1(ind2)=payoff1(ind2)+WPD(strategyPD(ind2),strategyPD(ind1));
        %strategies in game B
        sHD(ind1)=strategyHD(ind1,strategyPD(ind2));
        sHD(ind2)=strategyHD(ind2,strategyPD(ind1));
```

```
    %Payoff in game B
    payoff2(ind1)=payoff2(ind1)+WHD(sHD(ind1),sHD(ind2));
    payoff2(ind2)=payoff2(ind2)+WHD(sHD(ind2),sHD(ind1));
end
%total payoff
payofft=payoff1+payoff2+base*ones(N,1);
%recording of strategies.
strategy=(strategyPD-1)*4+(strategyHD(:,1)-1)*2+strategyHD(:,2)-1;
if mod(t,caltime)==0 && t¿=Ti
    tr=tr+1;
    rhoCdd(tr)=sum(strategy==0);
    rhoCdu(tr)=sum(strategy==1);
    rhoCud(tr)=sum(strategy==2);
    rhoCuu(tr)=sum(strategy==3);
    rhoDdd(tr)=sum(strategy==4);
    rhoDdu(tr)=sum(strategy==5);
    rhoDud(tr)=sum(strategy==6);
    rhoDuu(tr)=sum(strategy==7);
    mp(tr)=mean(payofft);
    rhod(tr)=mean(sHD==1);
    sPD=1-2*(strategyPD-1);
    sHD=1-2*(sHD-1);
    cor(tr)=mean((sHD-mean(sHD)).*(sPD-mean(sPD)));
    aq(1)=mean(payoff1(strategy==0));
    len1(1)=sum((strategy==0));
    aq(2)=mean(payoff1(strategy==1));
    len1(2)=sum((strategy==1));
    aq(3)=mean(payoff1(strategy==2));
    len1(3)=sum((strategy==2));
    aq(4)=mean(payoff1(strategy==3));
    len1(4)=sum((strategy==3));
    aq(isnan(aq))=0;
    piCA(tr)=sum(len1.*aq)/sum(len1);
    aq(1)=mean(payoff2(strategy==0));
    len1(1)=sum((strategy==0));
    aq(2)=mean(payoff2(strategy==1));
    len1(2)=sum((strategy==1));
    aq(3)=mean(payoff2(strategy==2));
    len1(3)=sum((strategy==2));
    aq(4)=mean(payoff2(strategy==3));
    len1(4)=sum((strategy==3));
    aq(isnan(aq))=0;
    pidB(tr)=sum(len1.*aq)/sum(len1);
    aq(1)=mean(payoff1(strategy==4));
    len1(1)=sum((strategy==4));
    aq(2)=mean(payoff1(strategy==5));
    len1(2)=sum((strategy==5));
    aq(3)=mean(payoff1(strategy==6));
```

```
    len1(3)=sum((strategy==6));
    aq(4)=mean(payoff1(strategy==7));
    len1(4)=sum((strategy==7));
    aq(isnan(aq))=0;
    piDA(tr)=sum(len1.*aq)/sum(len1);
    aq(1)=mean(payoff2(strategy==4));
    len1(1)=sum((strategy==4));
    aq(2)=mean(payoff2(strategy==5));
    len1(2)=sum((strategy==5));
    aq(3)=mean(payoff2(strategy==6));
    len1(3)=sum((strategy==6));
    aq(4)=mean(payoff2(strategy==7));
    len1(4)=sum((strategy==7));
    q(isnan(aq))=0;
    piuB(tr)=sum(len1.*aq)/sum(len1);
end
% selection step
strategyPD1=strategyPD;
strategyHD1=strategyHD;
payofft(payofft¡0)=0;
if sum(payofft)==0;
    payofft=ones(1,N);
end;
payofft=(payofft)/sum(payofft);
% The whole population is updated
for it=1:N;
    tesr=0;
    tesr2=rand(1);
    for ie=1:N;
        tesr=tesr+payofft(ie);
        if tesr >= tesr2;
            winner=ie;
            break;
        end;
    end;
    strategyPD1(it)=strategyPD(winner);
    strategyHD1(it,:)=strategyHD(winner,:);
    % mutation
    if nu1¿rand(1);
        a=randi(8)-1;
        while a==strategy(winner)
            a=randi(8)-1;
        end
        strategyPD1(it)=floor(a/4)+1;
        aa=mod(a,4);
        strategyHD1(it,1)=floor(aa/2)+1;
        strategyHD1(it,2)=mod(aa,2)+1;
    end
```

```
        end;
        strategyPD=strategyPD1;
        strategyHD=strategyHD1;
        payofft=0*ones(N,1);
        payoff1=0*ones(N,1);
        payoff2=0*ones(N,1);
    end
    rhoCdd=rhoCdd/N;rhoCdu=rhoCdu/N;rhoCud=rhoCud/N;rhoCuu=rhoCuu/N;
    rhoDdd=rhoDdd/N;rhoDdu=rhoDdu/N;rhoDud=rhoDud/N;rhoDuu=rhoDuu/N;
```

J.1.2 Numerical solution of the replicator dynamics

% This function is used for numerical solution of the replicator dynamics of the direct interaction model.
Output:
rhoCuut to rhoDddt are the density of different strategies (the same notation as in the main text is used). PiCA and piDA are the payoff of ,respectively, cooperators and defectors from the prisoner's dilemma, pidB and piuB are the payoffs of, respectively, down and up strategies from game B. mpit is the average payoff.
Input:
T2 is the duration of simulation, nu is the mutation rate. R, T, P, and S and the payoffs of the prisoner's dilemma. RB, TB, PB, and SB are the payoffs of game B, base is a base payoff, and caltime is the interval between recording the results. initial is a 1 X 8 vector of intiial conditions

```
    function [ rhoCuut , rhoCudt , rhoCdut , rhoCddt , rhoDuut , rhoDudt , rhoDdut , rhoDddt , piCA , piDA , pidB , piuB , mpit ] = fmfDirectInteraction ( T2 , nu , base , R , T , P , S , RB , TB , PB , SB , initial , caltime )
    %setting the initial conditions
    initial=initial/sum(initial);
    rhoCuu=initial(1);
    rhoCud=initial(2);
    rhoCdu=initial(3);
    rhoCdd=initial(4);
    rhoDuu=initial(5);
    rhoDud=initial(6);
    rhoDdu=initial(7);
    rhoDdd=initial(8);
    tr=0;
    for t=1:T2;
        rhoCHaC=rhoCuu+rhoCud;
        rhoDHaC=rhoDuu+rhoDud;
        rhoCdoC=rhoCdu+rhoCdd;
        rhoDdoC=rhoDdu+rhoDdd;
        rhoCHaD=rhoCuu+rhoCdu;
        rhoDHaD=rhoDuu+rhoDdu;
        rhoCdoD=rhoCud+rhoCdd;
        rhoDdoD=rhoDud+rhoDdd;
        % payoffs from the prisonner's dilemma
        piCuu1=rhoCHaC*(R)+rhoDHaC*(S)+rhoCdoC*(R)+rhoDdoC*(S);
```

```
piCud1=rhoCHaC*(R)+rhoDHaC*(S)+rhoCdoC*(R)+rhoDdoC*(S);
piCdu1=rhoCHaC*(R)+rhoDHaC*(S)+rhoCdoC*(R)+rhoDdoC*(S);
piCdd1=rhoCHaC*(R)+rhoDHaC*(S)+rhoCdoC*(R)+rhoDdoC*(S);
piDuu1=rhoCHaD*(T)+rhoDHaD*(P)+rhoCdoD*(T)+rhoDdoD*(P);
piDud1=rhoCHaD*(T)+rhoDHaD*(P)+rhoCdoD*(T)+rhoDdoD*(P);
piDdu1=rhoCHaD*(T)+rhoDHaD*(P)+rhoCdoD*(T)+rhoDdoD*(P);
piDdd1=rhoCHaD*(T)+rhoDHaD*(P)+rhoCdoD*(T)+rhoDdoD*(P);
% payoffs from game B
piCuu2=rhoCHaC*(PB)+rhoDHaC*(PB)+rhoCdoC*(TB)+rhoDdoC*(TB);
piCud2=rhoCHaC*(PB)+rhoDHaC*(SB)+rhoCdoC*(TB)+rhoDdoC*(RB);
piCdu2=rhoCHaC*(SB)+rhoDHaC*(PB)+rhoCdoC*(RB)+rhoDdoC*(TB);
piCdd2=rhoCHaC*(SB)+rhoDHaC*(SB)+rhoCdoC*(RB)+rhoDdoC*(RB);
piDuu2=rhoCHaD*(PB)+rhoDHaD*(PB)+rhoCdoD*(TB)+rhoDdoD*(TB);
piDud2=rhoCHaD*(PB)+rhoDHaD*(SB)+rhoCdoD*(TB)+rhoDdoD*(RB);
piDdu2=rhoCHaD*(SB)+rhoDHaD*(PB)+rhoCdoD*(RB)+rhoDdoD*(TB);
piDdd2=rhoCHaD*(SB)+rhoDHaD*(SB)+rhoCdoD*(RB)+rhoDdoD*(RB);
% total payoffs
piCuu=piCuu1+piCuu2+base;
piCud=piCud1+piCud2+base;
piCdu=piCdu1+piCdu2+base;
piCdd=piCdd1+piCdd2+base;
piDuu=piDuu1+piDuu2+base;
piDud=piDud1+piDud2+base;
piDdu=piDdu1+piDdu2+base;
piDdd=piDdd1+piDdd2+base;
% average payoff
mpi=rhoCuu*piCuu+rhoCud*piCud+rhoCdu*piCdu+rhoCdd*piCdd+...
rhoDuu*piDuu+rhoDud*piDud+rhoDdu*piDdu+rhoDdd*piDdd;
% updating the strategies (before mutation)
rhoCuu=(piCuu/mpi)*rhoCuu;
rhoCud=(piCud/mpi)*rhoCud;
rhoCdu=(piCdu/mpi)*rhoCdu;
rhoCdd=(piCdd/mpi)*rhoCdd;
rhoDuu=(piDuu/mpi)*rhoDuu;
rhoDud=(piDud/mpi)*rhoDud;
rhoDdu=(piDdu/mpi)*rhoDdu;
rhoDdd=(piDdd/mpi)*rhoDdd;
Bb=rhoCuu+rhoCud+rhoCdu+rhoCdd+rhoDuu+rhoDud+rhoDdu+rhoDdd;
% mutation is taken into account
rhoCuu=(1-nu)*rhoCuu+nu*(Bb-rhoCuu)/7;
rhoCud=(1-nu)*rhoCud+nu*(Bb-rhoCud)/7;
rhoCdu=(1-nu)*rhoCdu+nu*(Bb-rhoCdu)/7;
rhoCdd=(1-nu)*rhoCdd+nu*(Bb-rhoCdd)/7;
rhoDuu=(1-nu)*rhoDuu+nu*(Bb-rhoDuu)/7;
rhoDud=(1-nu)*rhoDud+nu*(Bb-rhoDud)/7;
rhoDdu=(1-nu)*rhoDdu+nu*(Bb-rhoDdu)/7;
rhoDdd=(1-nu)*rhoDdd+nu*(Bb-rhoDdd)/7;
% recording of data
```

```
    if mod(t,caltime)==0
        tr=tr+1;
        rhoCuut(tr)=rhoCuu;
        rhoCudt(tr)=rhoCud;
        rhoCdut(tr)=rhoCdu;
        rhoCddt(tr)=rhoCdd;
        rhoDuut(tr)=rhoDuu;
        rhoDudt(tr)=rhoDud;
        rhoDdut(tr)=rhoDdu;
        rhoDddt(tr)=rhoDdd;
        piCA(tr)=(rhoCuu*piCuu1+rhoCud*piCud1+rhoCdu*piCdu1+...
        rhoCdd*piCdd1)/(rhoCuu+rhoCud+rhoCdu+rhoCdd);
        piDA(tr)=(rhoDuu*piDuu1+rhoDuu*piDud1+rhoDdu*piDdu1+...
        rhoDdd*piDdd1)/(rhoDuu+rhoDuu+rhoDdu+rhoDdd);
        pidB(tr)=(rhoCuu*piCuu2+rhoCud*piCud2+rhoCdu*piCdu2+...
        rhoCdd*piCdd2)/(rhoCuu+rhoCud+rhoCdu+rhoCdd);
        piuB(tr)=(rhoDuu*piDuu2+rhoDud*piDud2+rhoDdu*piDdu2+...
        rhoDdd*piDdd2)/(rhoDuu+rhoDud+rhoDdu+rhoDdd);
        mpit(tr)=mpi;
    end
end
```

J.2 Codes for the Reputation-based model

J.2.1 Simulations

This function is used for the simulations of the direct interaction model.
Output:
rho Cuu to rho Ddd are the density of different strategies (the same notation as in the main text is used). PiCA and piDA are the payoff of ,respectively, cooperators and defectors from the prisoner's dilemma, pidB and piuB are the payoffs of, respectively, down and up strategies from game B. mp is the average payoff, rhod is the density of the strategy down, and cor is the correlations between the strategies in the prisoner's dilemma and game B.
Input:
T is the duration of simulation, Ti is the initial time of recording the simulation results, q is the probability of error in inferring the reputation of the opponent, N is the population size, nu1 is the mutation rate. Reward, Temptation, Punish, and Succer and the payoffs of the prisoner's dilemma. RewardB, TemptationB, PunishB, and SuccerB are the payoffs of game B, base is a base payoff, and caltime is the interval between recording the results.

```
function [ mCuu , mCud , mCdu , mCdd , mDuu , mDud , mDdu , mDdd , piCA , piDA , pidB , piuB , mp , mdo , cor ] = fReputationbased ( T , Ti , q , N , nu1 , Reward , Temptation , Punish , Succer , RewardHD , TemptationHD , PunishHD , SuccerHD , base , caltime )
    %setting the initial conditions for the prisoner's dilemma (strategyPD) and game B
    (strategyHD).
    strategyPD=randi(2,N,1);
    strategyHD=randi(2,N,2);
    sHD=zeros(N,1);
    % The strategy matrix of the prosiner's dilemma.
```

```
WPD=[Reward,Succer;Temptation,Punish];
% The strategy matrix of game B.
WHD=[RewardHD,SuccerHD;TemptationHD,PunishHD];
payoff1=0*ones(N,1);
payoff2=0*ones(N,1);
payoff=0*ones(N,1);
tr=0;
for t=1:T;
    % random pairing for playing the Prisoner's dilemma
    popvec=1:N;
    while ~isempty(popvec);
        Len=length(popvec);
        a1=randi(Len);
        ind1=popvec(a1);
        popvec(a1)=[];
        a2=randi(Len-1);
        ind2=popvec(a2);
        popvec(a2)=[];
        % payoff from the prisoner's dilemma
        payoff1(ind1)=payoff1(ind1)+WPD(strategyPD(ind1),strategyPD(ind2));
        payoff1(ind2)=payoff1(ind2)+WPD(strategyPD(ind2),strategyPD(ind1));
    end
    % random pairing for playing game B
    popvec=1:N;
    while ~isempty(popvec);
        Len=length(popvec);
        a1=randi(Len);
        ind1=popvec(a1);
        popvec(a1)=[];
        a2=randi(Len-1);
        ind2=popvec(a2);
        popvec(a2)=[];
        if q>rand(1);
            sHD(ind1)=strategyHD(ind1,3-strategyPD(ind2));
        else
            sHD(ind1)=strategyHD(ind1,strategyPD(ind2));
        end
        if q>rand(1);
            sHD(ind2)=strategyHD(ind2,3-strategyPD(ind1));
        else
            sHD(ind2)=strategyHD(ind2,strategyPD(ind1));
        end
        % payoff from game B
        payoff2(ind1)=payoff2(ind1)+WHD(sHD(ind1),sHD(ind2));
        payoff2(ind2)=payoff2(ind2)+WHD(sHD(ind2),sHD(ind1));
    end
    % total payoff
    payoff=payoff1+payoff2+base*ones(N,1);
```

```
% recording of output variables
strategy = (strategyPD-1)*4 + (strategyHD(:,1)-1)*2 + strategyHD(:,2)-1;
if mod(t,caltime)==0 && t¿Ti;
    tr=tr+1;
    mCdd(tr)=sum(strategy==0);
    mCdu(tr)=sum(strategy==1);
    mCud(tr)=sum(strategy==2);
    mCuu(tr)=sum(strategy==3);
    mDdd(tr)=sum(strategy==4);
    mDdu(tr)=sum(strategy==5);
    mDud(tr)=sum(strategy==6);
    mDuu(tr)=sum(strategy==7);
    mp(tr)=mean(payoff);
    mdo(tr)=mean(sHD==1);
    sPD=1-2*(strategyPD-1);
    sHD=1-2*(sHD-1);
    cor(tr)=mean((sHD-mean(sHD)).*(sPD-mean(sPD)));
    aq(1)=mean(payoff1(strategy==0));
    len1(1)=sum((strategy==0));
    aq(2)=mean(payoff1(strategy==1));
    len1(2)=sum((strategy==1));
    aq(3)=mean(payoff1(strategy==2));
    len1(3)=sum((strategy==2));
    aq(4)=mean(payoff1(strategy==3));
    len1(4)=sum((strategy==3));
    aq(isnan(aq))=0;
    piCA(tr)=sum(len1.*aq)/sum(len1);
    aq(1)=mean(payoff2(strategy==0));
    len1(1)=sum((strategy==0));
    aq(2)=mean(payoff2(strategy==1));
    len1(2)=sum((strategy==1));
    aq(3)=mean(payoff2(strategy==2));
    len1(3)=sum((strategy==2));
    aq(4)=mean(payoff2(strategy==3));
    len1(4)=sum((strategy==3));
    aq(isnan(aq))=0;
    pidB(tr)=sum(len1.*aq)/sum(len1);
    aq(1)=mean(payoff1(strategy==4));
    len1(1)=sum((strategy==4));
    aq(2)=mean(payoff1(strategy==5));
    len1(2)=sum((strategy==5));
    aq(3)=mean(payoff1(strategy==6));
    len1(3)=sum((strategy==6));
    aq(4)=mean(payoff1(strategy==7));
    len1(4)=sum((strategy==7));
    aq(isnan(aq))=0;
    piDA(tr)=sum(len1.*aq)/sum(len1);
    aq(1)=mean(payoff2(strategy==4));
```

```
    len1(1)=sum((strategy==4));
    aq(2)=mean(payoff2(strategy==5));
    len1(2)=sum((strategy==5));
    aq(3)=mean(payoff2(strategy==6));
    len1(3)=sum((strategy==6));
    aq(4)=mean(payoff2(strategy==7));
    len1(4)=sum((strategy==7));
    aq(isnan(aq))=0;
    piuB(tr)=sum(len1.*aq)/sum(len1);
end
% selection step
strategyPD1=strategyPD;
strategyHD1=strategyHD;
payoff(payoff¡0)=0;
if sum(payoff)==0;
    payoff=ones(1,N);
end;
payoff=(payoff)/sum(payoff);
% The whole population is updated.
for it=1:N;
    tesr=0;
    tesr2=rand(1);
    for ie=1:N;
        tesr=tesr+payoff(ie);
        if tesr ¿= tesr2;
            winner=ie;
            break;
        end;
    end;
    strategyPD1(it)=strategyPD(winner);
    strategyHD1(it,:)=strategyHD(winner,:);
    % mutation
    if nu1>rand(1);
        a=randi(8)-1;
        while a==strategy(winner)
            a=randi(8)-1;
        end
        strategyPD1(it)=floor(a/4)+1;
        aa=mod(a,4);
        strategyHD1(it,1)=floor(aa/2)+1;
        strategyHD1(it,2)=mod(aa,2)+1;
    end
end;
strategyPD=strategyPD1;
strategyHD=strategyHD1;
payoff=0*ones(N,1);
payoff1=0*ones(N,1);
payoff2=0*ones(N,1);
```

```
end
mCdd=mCdd/N;mCdu=mCdu/N;mCud=mCud/N;mCuu=mCuu/N;
mDdd=mDdd/N;mDdu=mDdu/N;mDud=mDud/N;mDuu=mDuu/N;
```

J.2.2 Numerical solution of the replicator dynamics

%This function is used for numerical solution of the replicator dynamics of the reputation-based model.

Output:

rhoCuut to rhoDddt are the density of different strategies (the same notation as in the main text is used). PiCA and piDA are the payoff of ,respectively, cooperators and defectors from the prisoner's dilemma, pidB and piuB are the payoffs of, respectively, down and up strategies from game B. mpit is the average payoff.

Input:

T2 is the duration of simulation, q is the error probability, nu is the mutation rate. R, T, P, and S and the payoffs of the prisoner's dilemma. RB, TB, PB, and SB are the payoffs of game B, base is a base payoff, and caltime is the interval between recording the results. initial is a 1 X 8 vector of intiial conditions

```
function [rhoCuut,rhoCudt,rhoCdut,rhoCddt,rhoDuut,... rhoDudt,rhoDdut,rhoDddt,piCA,piDA,pidB,piuB,mp
fmfReputationbased(T2,q,nu,base,R,T,P,S,RB,TB,PB,SB,initial,caltime)
    %setting the initial conditions
    initial=initial/sum(initial);
    rhoCuu=initial(1);
    rhoCud=initial(2);
    rhoCdu=initial(3);
    rhoCdd=initial(4);
    rhoDuu=initial(5);
    rhoDud=initial(6);
    rhoDdu=initial(7);
    rhoDdd=initial(8);
    tr=0;
    for t=1:T2;
        rhoCHaC=rhoCuu+rhoCud;
        rhoDHaC=rhoDuu+rhoDud;
        rhoCdoC=rhoCdu+rhoCdd;
        rhoDdoC=rhoDdu+rhoDdd;
        rhoCHaD=rhoCuu+rhoCdu;
        rhoDHaD=rhoDuu+rhoDdu;
        rhoCdoD=rhoCud+rhoCdd;
        rhoDdoD=rhoDud+rhoDdd;
        % payoffs from the prisonner's dilemma
        piCuu1=rhoCHaC*(R)+rhoDHaC*(S)+rhoCdoC*(R)+rhoDdoC*(S);
        piCud1=rhoCHaC*(R)+rhoDHaC*(S)+rhoCdoC*(R)+rhoDdoC*(S);
        piCdu1=rhoCHaC*(R)+rhoDHaC*(S)+rhoCdoC*(R)+rhoDdoC*(S);
        piCdd1=rhoCHaC*(R)+rhoDHaC*(S)+rhoCdoC*(R)+rhoDdoC*(S);
        piDuu1=rhoCHaD*(T)+rhoDHaD*(P)+rhoCdoD*(T)+rhoDdoD*(P);
        piDud1=rhoCHaD*(T)+rhoDHaD*(P)+rhoCdoD*(T)+rhoDdoD*(P);
        piDdu1=rhoCHaD*(T)+rhoDHaD*(P)+rhoCdoD*(T)+rhoDdoD*(P);
```

```
piDdd1=rhoCHaD*(T)+rhoDHaD*(P)+rhoCdoD*(T)+rhoDdoD*(P);
% payoffs from game B
piCuu2=rhoCuu*PB+(1-q)*rhoCud*(PB)+q*rhoCud*TB+...
rhoDuu*(PB)+(1-q)*rhoDud*PB+q*rhoDud*TB+...
rhoCdd*(TB)+(1-q)*rhoCdu*TB+q*rhoCdu*PB+...
rhoDdd*(TB)+(1-q)*rhoDdu*TB+q*rhoDdu*PB;
piCud2=(1-q)*(rhoCuu*PB+(1-q)*rhoCud*(PB)+q*rhoCud*TB)+...
q*(rhoCuu*SB+(1-q)*rhoCud*(SB)+q*rhoCud*RB)+...
(1-q)*(rhoDuu*(SB)+(1-q)*rhoDud*SB+q*rhoDud*RB)+...
q*(rhoDuu*(PB)+(1-q)*rhoDud*PB+q*rhoDud*TB)+...
(1-q)*(rhoCdd*(TB)+(1-q)*rhoCdu*TB+q*rhoCdu*PB)+...
q*(rhoCdd*(RB)+(1-q)*rhoCdu*RB+q*rhoCdu*SB)+...
+(1-q)*(rhoDdd*(RB)+(1-q)*rhoDdu*RB+q*rhoDdu*SB)+...
q*(rhoDdd*(TB)+(1-q)*rhoDdu*TB+q*rhoDdu*PB);
piCdu2=(1-q)*(rhoCuu*SB+(1-q)*rhoCud*(SB)+q*rhoCud*RB)+...
q*(rhoCuu*PB+(1-q)*rhoCud*(PB)+q*rhoCud*TB)+...
(1-q)*(rhoDuu*(PB)+(1-q)*rhoDud*PB+q*rhoDud*TB)+...
q*(rhoDuu*(SB)+(1-q)*rhoDud*SB+q*rhoDud*RB)+...
(1-q)*(rhoCdd*(RB)+(1-q)*rhoCdu*RB+q*rhoCdu*SB)+...
q*(rhoCdd*(TB)+(1-q)*rhoCdu*TB+q*rhoCdu*PB)+...
(1-q)*(rhoDdd*(TB)+(1-q)*rhoDdu*TB+q*rhoDdu*PB)+...
q*(rhoDdd*(RB)+(1-q)*rhoDdu*RB+q*rhoDdu*SB);
piCdd2=rhoCuu*SB+(1-q)*rhoCud*(SB)+q*rhoCud*RB+...
rhoDuu*(SB)+(1-q)*rhoDud*SB+q*rhoDud*RB+...
(rhoCdd*(RB)+(1-q)*rhoCdu*RB+q*rhoCdu*SB)+...
rhoDdd*(RB)+(1-q)*rhoDdu*RB+q*rhoDdu*SB;
piDuu2=rhoCuu*PB+(1-q)*rhoCud*(TB)+q*rhoCud*PB+...
rhoDuu*(PB)+(1-q)*rhoDud*TB+q*rhoDud*PB+...
rhoCdd*(TB)+(1-q)*rhoCdu*PB+q*rhoCdu*TB+...
rhoDdd*(TB)+(1-q)*rhoDdu*PB+q*rhoDdu*TB;
piDud2=(1-q)*(rhoCuu*PB+(1-q)*rhoCud*(TB)+q*rhoCud*PB)+...
q*(rhoCuu*SB+(1-q)*rhoCud*(RB)+q*rhoCud*SB)+...
(1-q)*(rhoDuu*(SB)+(1-q)*rhoDud*RB+q*rhoDud*SB)+...
q*(rhoDuu*(PB)+(1-q)*rhoDud*TB+q*rhoDud*PB)+...
(1-q)*(rhoCdd*(TB)+(1-q)*rhoCdu*PB+q*rhoCdu*TB)+...
q*(rhoCdd*(RB)+(1-q)*rhoCdu*SB+q*rhoCdu*RB)+...
+(1-q)*(rhoDdd*(RB)+(1-q)*rhoDdu*SB+q*rhoDdu*RB)+...
q*(rhoDdd*(TB)+(1-q)*rhoDdu*PB+q*rhoDdu*TB);
piDdu2=(1-q)*(rhoCuu*SB+(1-q)*rhoCud*(RB)+q*rhoCud*SB)+...
q*(rhoCuu*PB+(1-q)*rhoCud*(TB)+q*rhoCud*PB)+...
(1-q)*(rhoDuu*(PB)+(1-q)*rhoDud*TB+q*rhoDud*PB)+...
q*(rhoDuu*(SB)+(1-q)*rhoDud*RB+q*rhoDud*SB)+...
(1-q)*(rhoCdd*(RB)+(1-q)*rhoCdu*SB+q*rhoCdu*RB)+...
q*(rhoCdd*(TB)+(1-q)*rhoCdu*PB+q*rhoCdu*TB)+...
(1-q)*(rhoDdd*(TB)+(1-q)*rhoDdu*PB+q*rhoDdu*TB)+...
q*(rhoDdd*(RB)+(1-q)*rhoDdu*SB+q*rhoDdu*RB);
piDdd2=rhoCuu*SB+(1-q)*rhoCud*(RB)+q*rhoCud*SB+...
rhoDuu*(SB)+(1-q)*rhoDud*RB+q*rhoDud*SB+...
```

```
(rhoCdd*(RB)+(1-q)*rhoCdu*SB+q*rhoCdu*RB)+...
rhoDdd*(RB)+(1-q)*rhoDdu*SB+q*rhoDdu*RB;
% total payoffs
piCuu=piCuu1+piCuu2+base;
piCud=piCud1+piCud2+base;
piCdu=piCdu1+piCdu2+base;
piCdd=piCdd1+piCdd2+base;
piDuu=piDuu1+piDuu2+base;
piDud=piDud1+piDud2+base;
piDdu=piDdu1+piDdu2+base;
piDdd=piDdd1+piDdd2+base;
% average payoff
mpi=rhoCuu*piCuu+rhoCud*piCud+rhoCdu*piCdu+rhoCdd*piCdd+...
rhoDuu*piDuu+rhoDud*piDud+rhoDdu*piDdu+rhoDdd*piDdd;
% updating the strategies (before mutation)
rhoCuu=(piCuu/mpi)*rhoCuu;
rhoCud=(piCud/mpi)*rhoCud;
rhoCdu=(piCdu/mpi)*rhoCdu;
rhoCdd=(piCdd/mpi)*rhoCdd;
rhoDuu=(piDuu/mpi)*rhoDuu;
rhoDud=(piDud/mpi)*rhoDud;
rhoDdu=(piDdu/mpi)*rhoDdu;
rhoDdd=(piDdd/mpi)*rhoDdd;
Bb=rhoCuu+rhoCud+rhoCdu+rhoCdd+rhoDuu+rhoDud+rhoDdu+rhoDdd;
% mutation is taken into account
rhoCuu=(1-nu)*rhoCuu+nu*(Bb-rhoCuu)/7;
rhoCud=(1-nu)*rhoCud+nu*(Bb-rhoCud)/7;
rhoCdu=(1-nu)*rhoCdu+nu*(Bb-rhoCdu)/7;
rhoCdd=(1-nu)*rhoCdd+nu*(Bb-rhoCdd)/7;
rhoDuu=(1-nu)*rhoDuu+nu*(Bb-rhoDuu)/7;
rhoDud=(1-nu)*rhoDud+nu*(Bb-rhoDud)/7;
rhoDdu=(1-nu)*rhoDdu+nu*(Bb-rhoDdu)/7;
rhoDdd=(1-nu)*rhoDdd+nu*(Bb-rhoDdd)/7;
% recording of data
if mod(t,caltime)==0
   tr=tr+1;
   rhoCuut(tr)=rhoCuu;
   rhoCudt(tr)=rhoCud;
   rhoCdut(tr)=rhoCdu;
   rhoCddt(tr)=rhoCdd;
   rhoDuut(tr)=rhoDuu;
   rhoDudt(tr)=rhoDud;
   rhoDdut(tr)=rhoDdu;
   rhoDddt(tr)=rhoDdd;
   piCA(tr)=(rhoCuu*piCuu1+rhoCud*piCud1+rhoCdu*piCdu1+...
   rhoCdd*piCdd1)/(rhoCuu+rhoCud+rhoCdu+rhoCdd);
   piDA(tr)=(rhoDuu*piDuu1+rhoDuu*piDud1+rhoDdu*piDdu1+...
   rhoDdd*piDdd1)/(rhoDuu+rhoDuu+rhoDdu+rhoDdd);
```

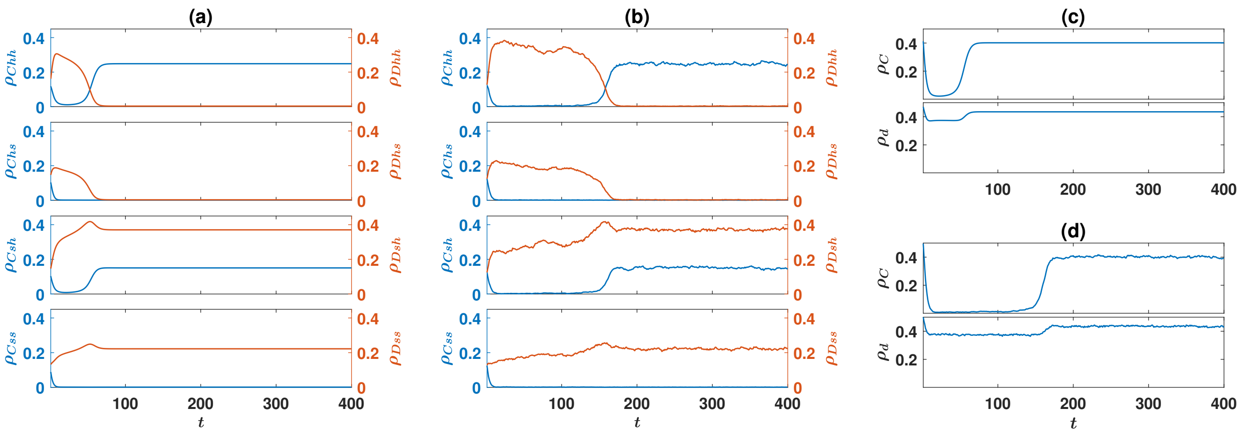

Figure A: Time evolution of the direct interaction model when game B is the Battle of the Sexes. (a) and (b): The time evolution of different strategies, resulted from the replicator dynamics (a), and a simulation in finite a population (b). (c) and (d): The time evolution of the density of the cooperators ρ_C (up), and the density of the soft strategies ρ_d (bottom), resulted from the replicator dynamics (c), and a simulation (d). The simulation is performed on a population of size $N = 20000$ and we have set $\nu = 0.005$. The initial condition is a random assignment of strategies (for the replicator dynamics this implies $\rho_x = 1/8$, for all strategies x). Here, the game B is the Battle of the Sexes and the payoff values shown in Table. B are used.

```
            pidB(tr)=(rhoCuu*piCuu2+rhoCud*piCud2+rhoCdu*piCdu2+...
            rhoCdd*piCdd2)/(rhoCuu+rhoCud+rhoCdu+rhoCdd);
            piuB(tr)=(rhoDuu*piDuu2+rhoDud*piDud2+rhoDdu*piDdu2+...
            rhoDdd*piDdd2)/(rhoDuu+rhoDud+rhoDdu+rhoDdd);
            mpit(tr)=mpi;
        end
    end
```

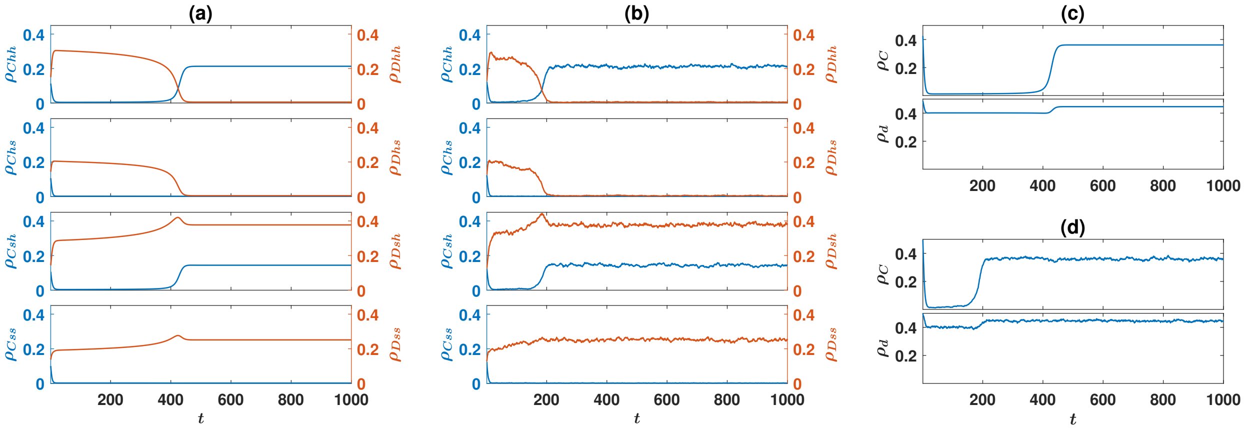

Figure B: Time evolution of the direct interaction model when game B is the Leader game. (a) and (b): The time evolution of different strategies, resulted from the replicator dynamics (a), and a simulation in finite a population (b). (c) and (d): The time evolution of the density of the cooperators ρ_C (up), and the density of the soft strategies ρ_d (bottom), resulted from the replicator dynamics (c), and a simulation (d). The simulation is performed on a population of size $N = 20000$ and we have set $\nu = 0.005$. The initial condition is a random assignment of strategies (for the replicator dynamics this implies $\rho_x = 1/8$, for all strategies x). Here, the game B is the Leader game and the payoff values shown in Table. B are used.

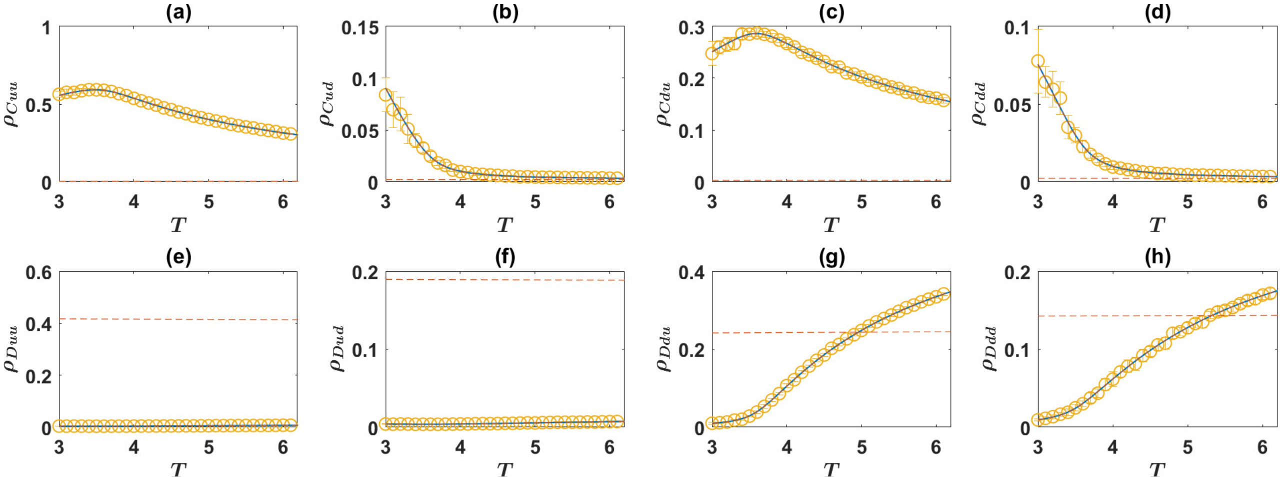

Figure C: The density of different strategies in the direct interaction model as a function of the temptation, T, when game B is the Snow Drift game. The density of different strategies as a function of the temptation, T, are plotted. The lines show the result of the replicator dynamics, and the markers show the results of simulations. Solid blue line shows the equilibrium fixed point, which occurs starting from an unbiased initial condition in which the density of all the strategies are equal, and the dashed red line shows the non-equilibrium fixed point, which can occur for certain initial conditions. For the simulations, a sample of 80 simulations, in a population of size $N = 10000$ is used. The simulations start from random initial conditions. In each simulation, the dynamics settle in one of the two fixed points. The markers show the averages, and the error bars show the standard deviation in the sample of simulations which settle in a given fixed point, and the size of markers is proportional to the number of times that the given fixed point occurs in the sample. Here, $\nu = 0.005$. The simulations are run for 20000 time steps, and an average over the last 1000 time steps is taken. Except for T, the payoff values shown in Table. B are used.

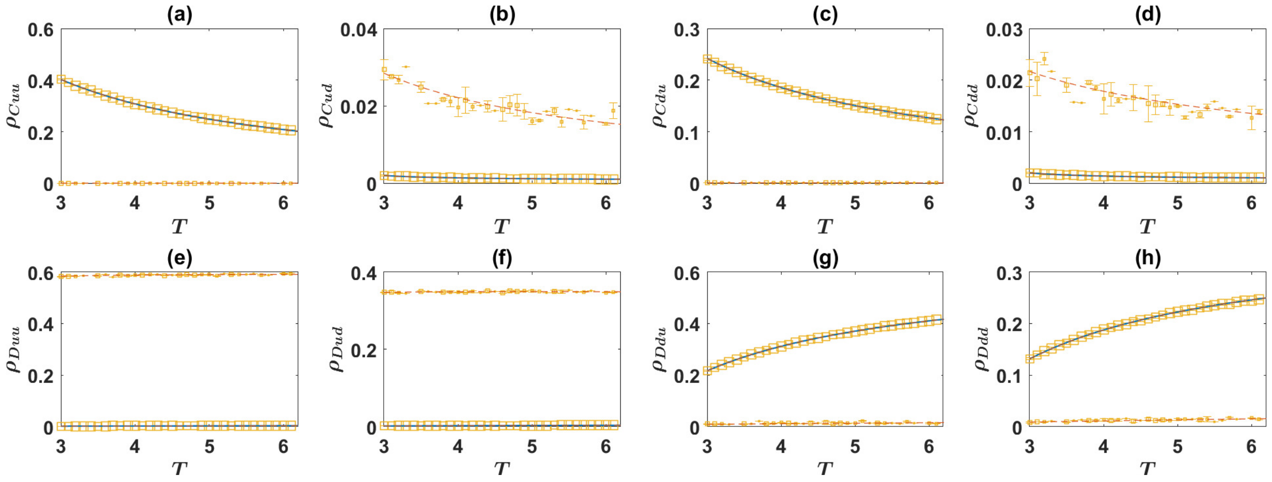

Figure D: The density of different strategies in the direct interaction model as a function of the temptation, T, when game B is the Battle of the Sexes. The density of different strategies as a function of the temptation, T, are plotted. The lines show the result of the replicator dynamics, and the markers show the results of simulations. Solid blue line shows the equilibrium fixed point, which occurs starting from an unbiased initial condition in which the density of all the strategies are equal, and the dashed red line shows the non-equilibrium fixed point, which can occur for certain initial conditions. For the simulations, a sample of 80 simulations, in a population of size $N = 10000$ is used. The simulations start from random initial conditions. In each simulation, the dynamics settle in one of the two fixed points. The markers show the averages, and the error bars show the standard deviation in the sample of simulations which settle in a given fixed point, and the size of markers is proportional to the number of times that the given fixed point occurs in the sample. Here, $\nu = 0.005$. The simulations are run for 20000 time steps, and an average over the last 1000 time steps is taken. Except for T, the payoff values shown in Table. B are used.

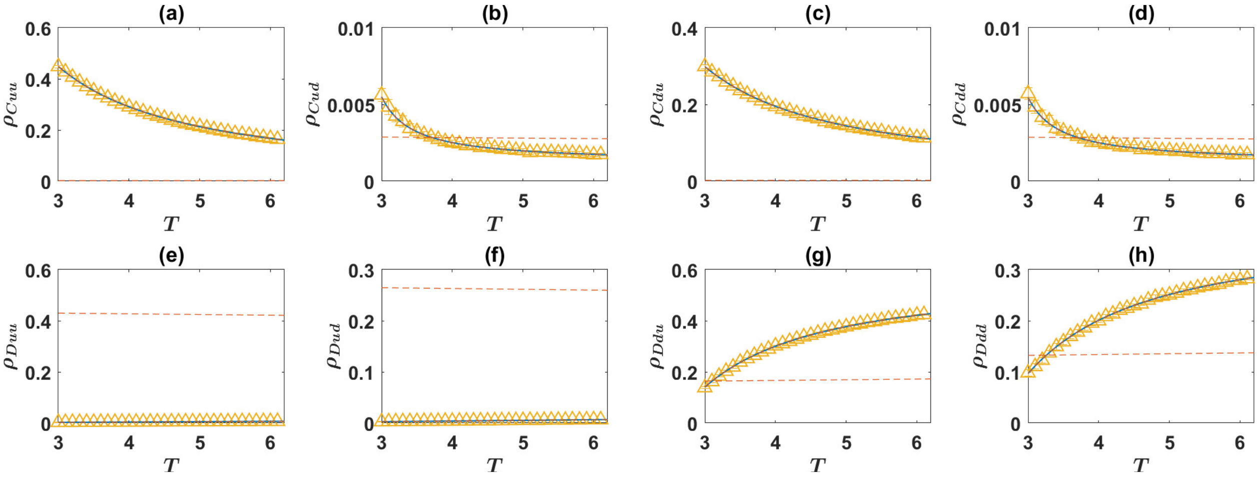

Figure E: The density of different strategies in the direct interaction model as a function of the temptation, T, when game B is the Leader game. The density of different strategies as a function of the temptation, T, are plotted. The lines show the result of the replicator dynamics, and the markers show the results of simulations. Solid blue line shows the equilibrium fixed point, which occurs starting from an unbiased initial condition in which the density of all the strategies are equal, and the dotted red line shows the non-equilibrium fixed point, which can occur for certain initial conditions. For the simulations, a sample of 80 simulations, in a population of size $N = 10000$ is used. The simulations start from random initial conditions. In each simulation, the dynamics settle in one of the two fixed points. The markers show the averages, and the error bars show the standard deviation in the sample of simulations which settle in a given fixed point, and the size of markers is proportional to the number of times that the given fixed point occurs in the sample. Here, $\nu = 0.005$. The simulations are run for 20000 time steps, and an average over the last 1000 time steps is taken. Except for T, the payoff values shown in Table. B are used.

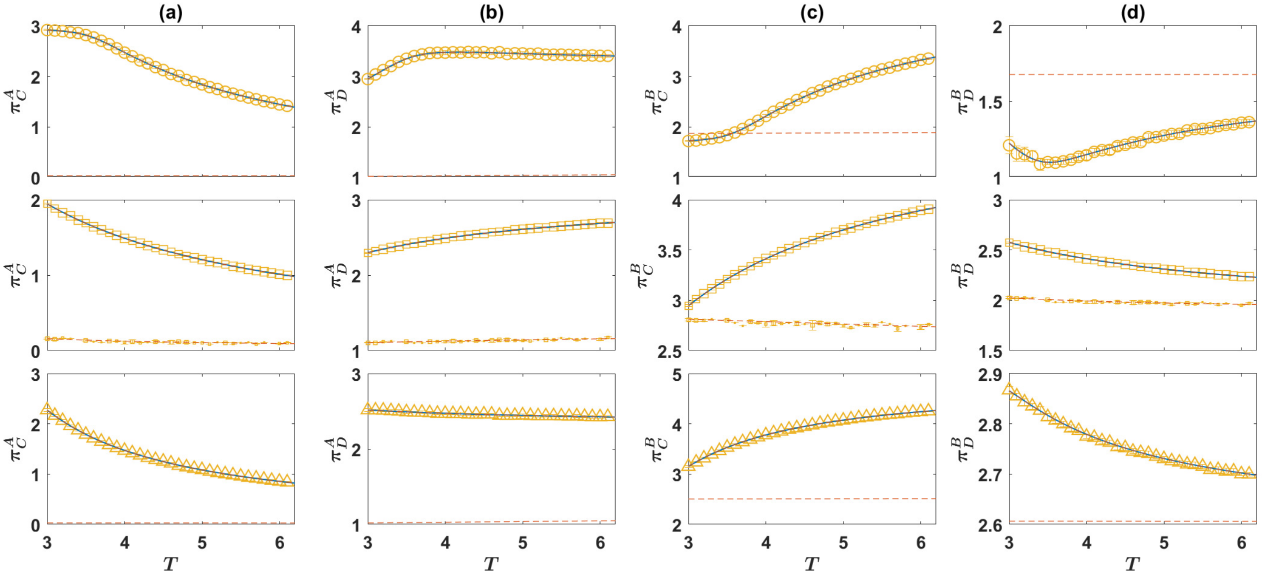

Figure F: The payoff of cooperators and defectors in the first game (A) and the second game (B), in the direct interaction model. Here, from top to bottom, game B is the Snow Drift, the Battle of the Sexes, and the Leader game. The payoffs are plotted as a function of the temptation, T. The lines show the result of the replicator dynamics, and the markers show the results of simulations. Solid blue line shows the equilibrium fixed point, which occurs starting from an unbiased initial condition in which the density of all the strategies are equal, and the dashed red line shows the non-equilibrium fixed point, which can occur for certain initial conditions. For the simulations, a sample of 80 simulations in a population of size $N = 10000$ is used. The simulations start from random initial conditions. In each simulation, the dynamics settle in one of the two fixed points. The markers show the averages, and the error bars show the standard deviation in the sample of simulations which settle in the given fixed point, and the size of markers is proportional to the number of times that the given fixed point occurs. Here, $\nu = 0.005$. The simulations are run for 20000 time steps, and an average over the last 1000 time steps is taken. Except for T, the payoff values shown in Table. B are used.

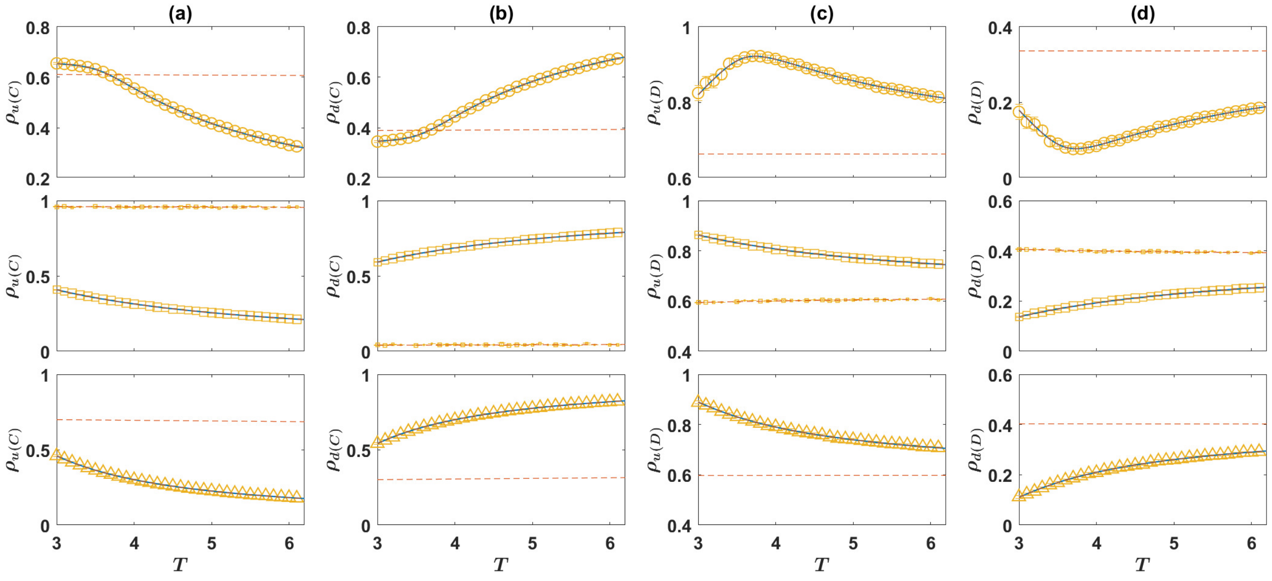

Figure G: Strategic response of cooperators and defectors in game B, in the direct interaction model. Here, from top to bottom, game B is the Snow Drift, the Battle of the Sexes, and the Leader game. The density of strategies who play up with cooperators $u(C)$, down with cooperators $d(C)$, up with defectors, $u(D)$, and down with defectors, $d(D)$, are plotted as a function of the temptation, T. The lines show the result of the replicator dynamics, and the markers show the results of simulations. Solid blue line shows the equilibrium fixed point, which occurs starting from an unbiased initial condition in which the density of all the strategies are equal, and the dashed red line shows the non-equilibrium fixed point, which can occur for certain initial conditions. For the simulations, a sample of 80 simulations, in a population of size $N = 10000$ is used. The simulations start from random initial conditions. In each simulation, the dynamics settle in one of the two fixed points. The markers show the averages, and the error bars show the standard deviation in the sample of simulations which settle in the given fixed point, and the size of markers is proportional to the number of times that the given fixed point occurs. Here, $\nu = 0.005$. The simulations are run for 20000 time steps, and an average over the last 1000 time steps is taken. Except for T, the payoff values shown in Table. B are used.

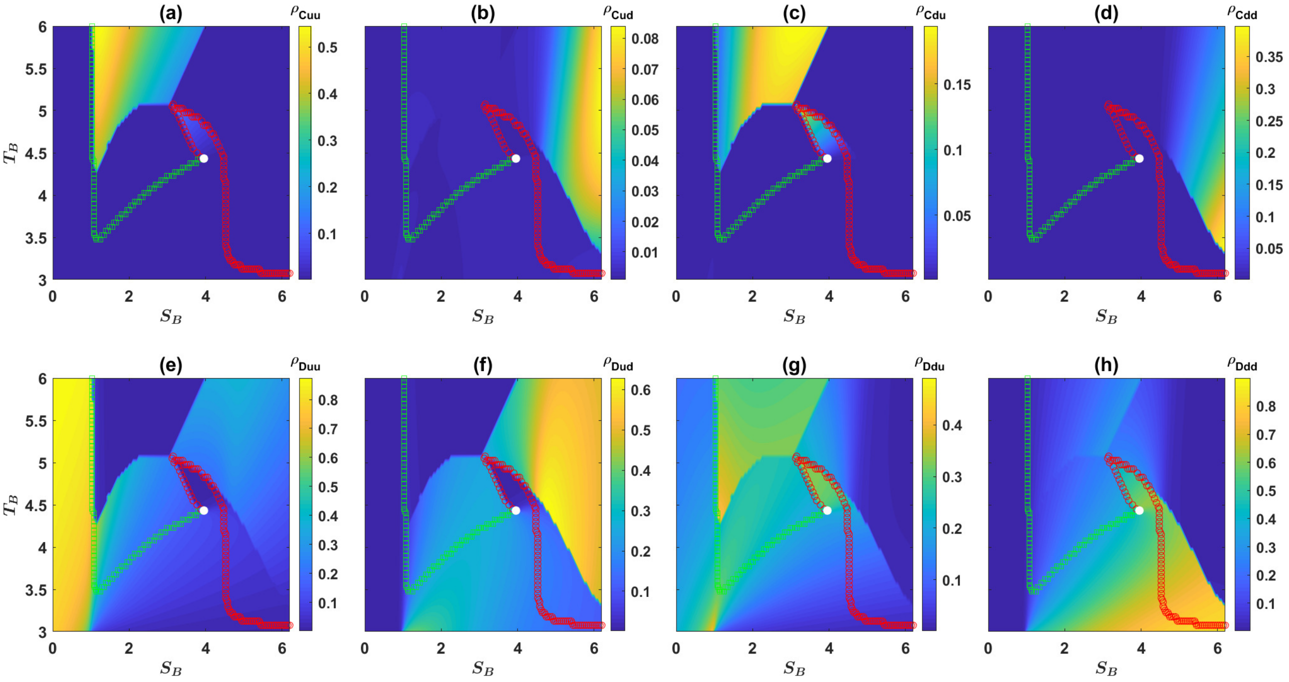

Figure H: The density of different strategies in the direct interaction model in $S_B - T_B$ plane. The density of different strategies, as indicated in the figure, are color plotted in $S_B - T_B$ plane. Here, starting from an unbiased initial condition in which the initial density of all the strategies is equal, the replicator dynamics is numerically solved. The boundaries of bistability are plotted as well. Below this boundary the dynamics is mono-stable, settling into a defective fixed point with a low level of cooperation. Above the boundary, a cooperative fixed point becomes stable and the dynamics become bistable. The two branches of the boundary meet at a critical point, where the transition becomes continuous. In the cooperative fixed point, cooperators always play hard with defectors and defectors play soft with cooperators. Here, $\nu = 0.005$. Except for T_B and S_B, the payoff values shown in Table. B are used.

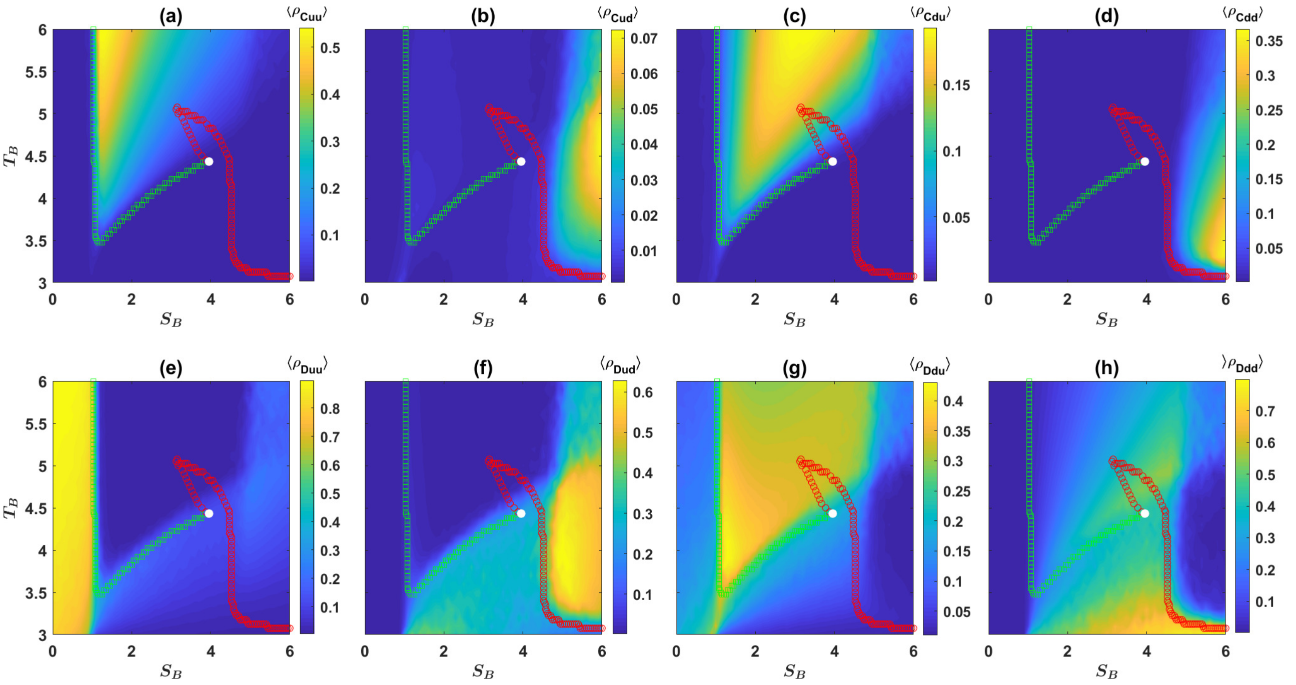

Figure I: The density of different strategies in the direct interaction model. The average density of different strategies (as indicated in the figure) over a sample of 128 simulations in a population of size 1000 individuals are color plotted in $S_B - T_B$ plane. The simulations start from random initial conditions in which the strategy the individuals are randomly assigned. The boundaries of bistability, derived from the replicator dynamics, are super-imposed in the figure as well. In a small population size, starting from a random initial condition, in the bistable region the dynamics settle in the cooperative fixed point with a high probability. Comparison with the results of replicator dynamics in H shows finite size effects favor cooperation. Here, $\nu = 0.005$. The simulations are run for 10000 time steps, and an average over the last 1000 time steps is taken. Except for T_B and S_B, the payoff values shown in Table. B are used.

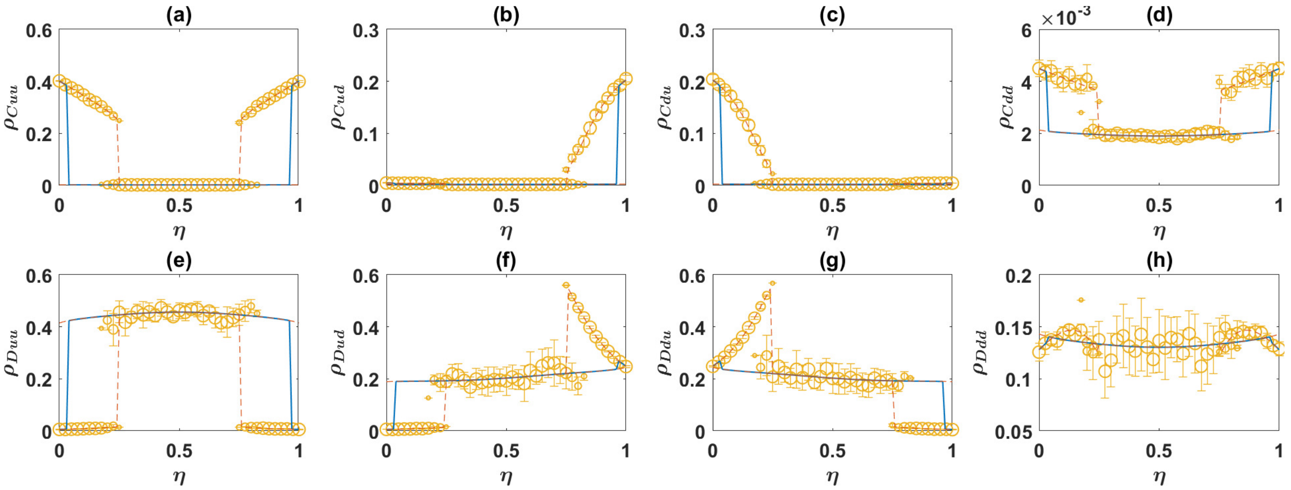

Figure J: The density of different strategies in the reputation-based model when game B is the Snow Drift game. The densities of different strategies as a function of the probability of error in inferring the reputation of the opponent, η, are plotted. The lines show the result of the replicator dynamics, and the markers show the results of simulations. Solid blue line shows the equilibrium fixed point, which occurs starting from an unbiased initial condition in which the density of all the strategies are equal, and the dashed red line shows the non-equilibrium fixed point, which can occur for certain initial conditions. For the simulations, a sample of 80 simulations in a population of size $N = 10000$ is used. The simulations start from random initial conditions. In each simulation, the dynamics settle in one of the two fixed points. The markers show the averages, and the error bars show the standard deviation in the sample of simulations which settle in a given fixed point, and the size of markers is proportional to the number of times that the given fixed point occurs. Comparison of the results of the replicator dynamics and simulations show finite size effects strongly favor cooperation. Here, $\nu = 0.005$. The simulations are run for 20000 time steps, and an average over the last 1000 time steps is taken. The payoff values shown in Table. B are used.

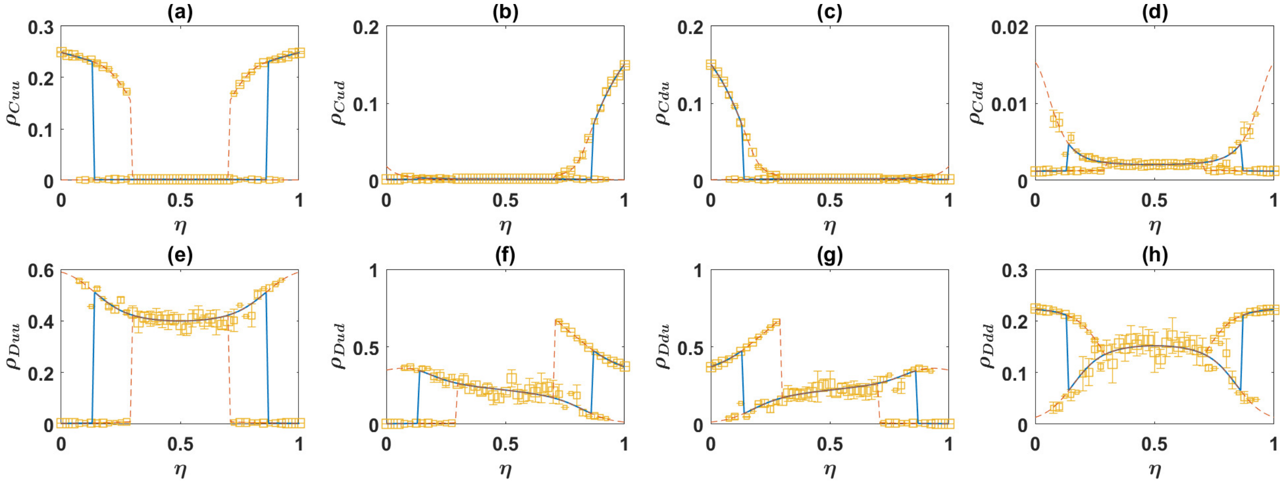

Figure K: The density of different strategies in the reputation-based model when game B is the Battle of the Sexes. The densities of different strategies as a function of the probability of error in inferring the reputation of the opponent, η, are plotted. The lines show the result of the replicator dynamics, and the markers show the results of simulations. Solid blue line shows the equilibrium fixed point, which occurs starting from an unbiased initial condition in which the density of all the strategies are equal, and the dashed red line shows the non-equilibrium fixed point, which can occur for certain initial conditions. For the simulations, a sample of 80 simulations in a population of size $N = 10000$ is used. The simulations start from random initial conditions. In each simulation, the dynamics settle in one of the two fixed points. The markers show the averages, and the error bars show the standard deviation in the sample of simulations which settle in a given fixed point, and the size of markers is proportional to the number of times that the given fixed point occurs. Comparison of the results of the replicator dynamics and simulations show finite size effects strongly favor cooperation. Here, $\nu = 0.005$. The simulations are run for 20000 time steps, and an average over the last 1000 time steps is taken. The payoff values shown in Table. B are used.

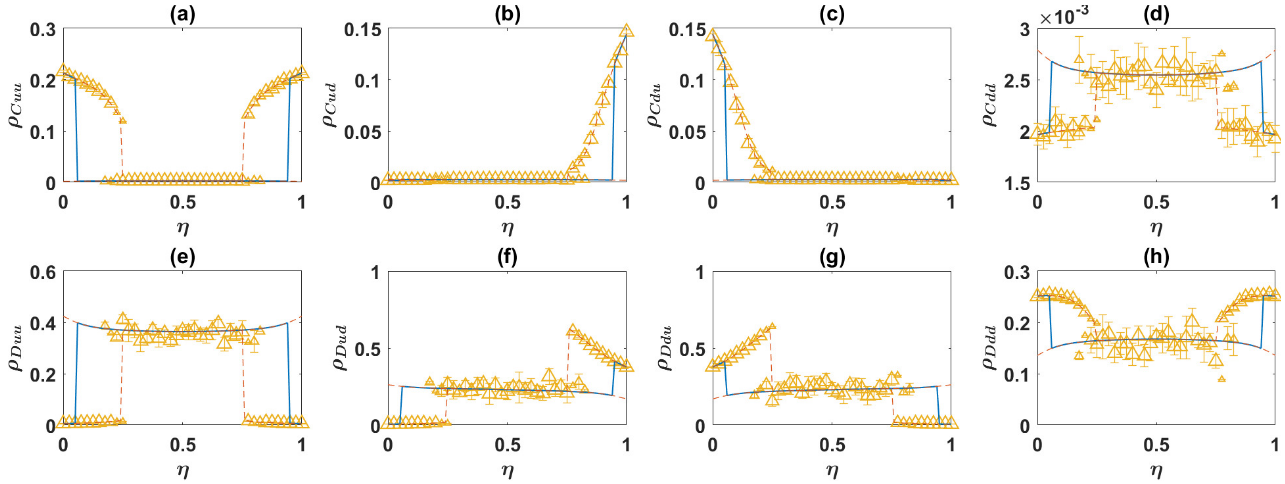

Figure L: The density of different strategies in the reputation-based model when game B is the Leader game. The densities of different strategies as a function of the probability of error in inferring the reputation of the opponent, η, are plotted. The lines show the result of the replicator dynamics, and the markers show the results of simulations. Solid blue line shows the equilibrium fixed point, which occurs starting from an unbiased initial condition in which the density of all the strategies are equal, and the dashed red line shows the non-equilibrium fixed point, which can occur for certain initial conditions. For the simulations, a sample of 80 simulations in a population of size $N = 10000$ is used. The simulations start from random initial conditions. In each simulation, the dynamics settle in one of the two fixed points. The markers show the averages, and the error bars show the standard deviation in the sample of simulations which settle in a given fixed point, and the size of markers is proportional to the number of times that the given fixed point occurs. Comparison of the results of the replicator dynamics and simulations show finite size effects strongly favor cooperation. Here, $\nu = 0.005$. The simulations are run for 20000 time steps, and an average over the last 1000 time steps is taken. The payoff values shown in Table. B are used.

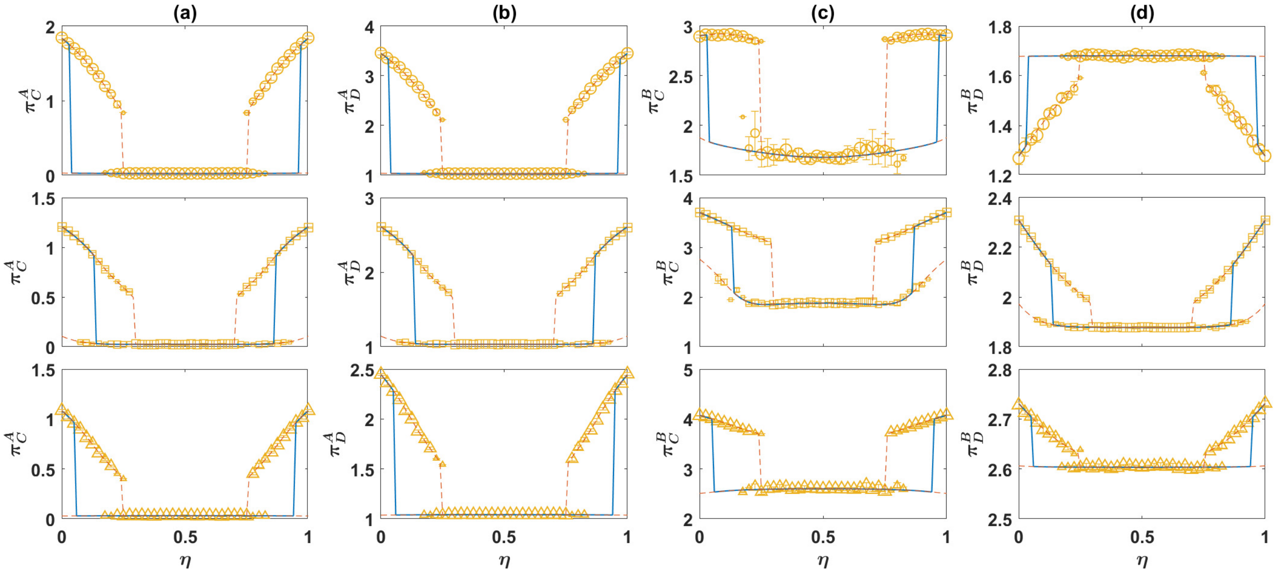

Figure M: The payoff of cooperators and defectors in the first game (A) and the second game (B), in the reputation-based model. Here, from top to bottom, game B is the Snow Drift, the Battle of the Sexes, and the Leader game. The payoffs are plotted as a function of the probability of error in inferring the reputation of the opponent, η. The lines show the result of the replicator dynamics, and the markers show the results of simulations. Solid blue line shows the equilibrium fixed point, which occurs starting from an unbiased initial condition in which the density of all the strategies are equal, and the dashed red line shows the non-equilibrium fixed point, which can occur for certain initial conditions. For the simulations, a sample of 80 simulations, in a population of size $N = 10000$ is used. The simulations start from random initial conditions. In each simulation, the dynamics settle in one of the two fixed points. The markers show the averages, and the error bars show the standard deviation in the sample of simulations which settle in the given fixed point, and the size of markers is proportional to the number of times that the given fixed point occurs. Comparison of the results of the replicator dynamics and simulations show finite size effects strongly favor cooperation. Here, $\nu = 0.005$. The simulations are run for 20000 time steps, and an average over the last 1000 time steps is taken. The payoff values shown in Table. B are used.

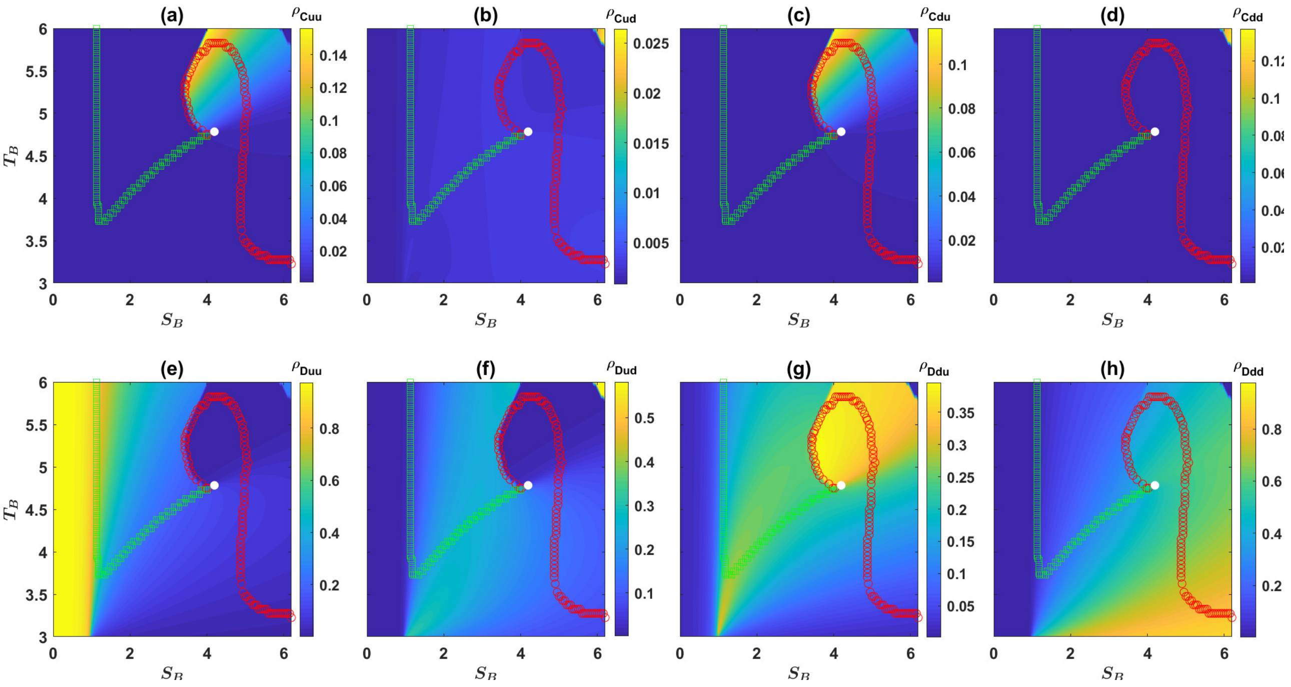

Figure N: The density of different strategies in the reputation-based model in the $S_B - T_B$ plane. The density of different strategies are color plotted in $S_B - T_B$ plane. Here, the replicator dynamics is numerically solved, starting from an unbiased initial condition in which the initial density of all the strategies is equal The boundaries of bistability are plotted as well. Below this boundary the dynamics is mono-stable, settling into a defective fixed point with a low level of cooperation. Above the boundary, a cooperative fixed point becomes stable and the dynamics become bistable. The two branches of the boundary meet at a critical point, where the transition becomes continuous. In the cooperative fixed point, cooperators always play hard with defectors and defectors play soft with cooperators. Here, $\eta = 0.1$, and $\nu = 0.005$. Payoff values: $S = 0$, $R = 3$, $P = 1$, $T = 5$, $R_B = 3$, $P_B = 1$.

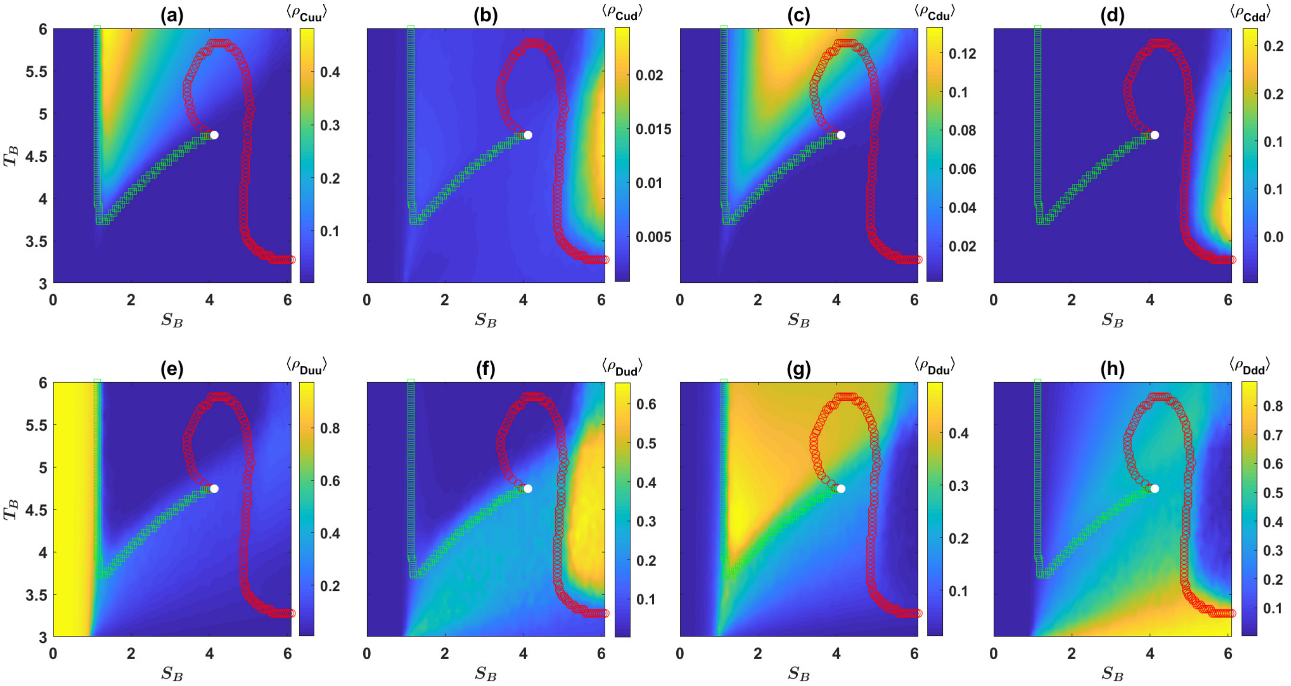

Figure O: The density of different strategies in the direct interaction model in the $S_B - T_B$ plane. The average density of different strategies (as indicated in the figure) over a sample of 128 simulations in a population of size 1000 individuals are color plotted in $S_B - T_B$ plane. The simulations start from random initial conditions. The boundaries of bistability, derived from the replicator dynamics, are super-imposed in the figure as well. In a small population size, starting from a random initial condition, in the bistable region the dynamics settle in the cooperative fixed point. Comparison with the results of replicator dynamics in N shows finite size effects favor cooperation. Here, $\nu = 0.005$. The simulations are run for 10000 time steps, and an average over the last 1000 time steps is taken. Payoff values: $S = 0$, $R = 3$, $P = 1$, $T = 5$, $R_B = 3$, $P_B = 1$.

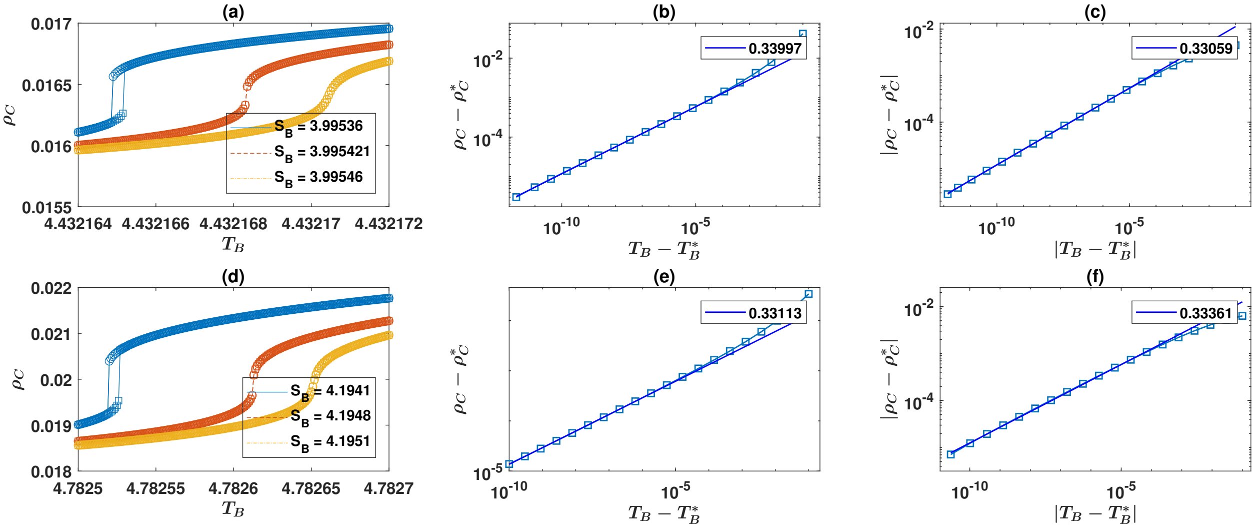

Figure P: The continuous phase transition. (a) and (d): The density of cooperators, starting from a cooperation favoring (circles) and defection favoring (squares) initial condition in the direct interaction (a) and reputation-based (d) models, as a function of T_B for three different values of S_B, chosen close to the critical point. In both models, the transition between the defective and the cooperative fixed points becomes a critical phase transition at a critical point (red dashed). For S_B smaller than the critical value (blue solid), by increasing T_B, the transition between the two fixed points is discontinuous and possesses a bistable region. For S_B larger than the critical value (dotted orange) by increasing T_B there is a cross-over from the defective to the cooperative fixed point. (b) and (c): In the direct interaction model, close the critical point the reduced order parameter as a function of the reduced control parameter shows a power-law relation for both above (b) and below (c) the critical point. (e) and (f): A similar scaling, with the same critical exponent is observed for the reputation-based model, above (e) and below (f) the phase transition. In all the cases, the replicator dynamics is solved numerically, setting $\nu = 0.005$, and and adding a base fitness of $b = 0.01$ to the individual. The payoff values shown in Table. B are used.

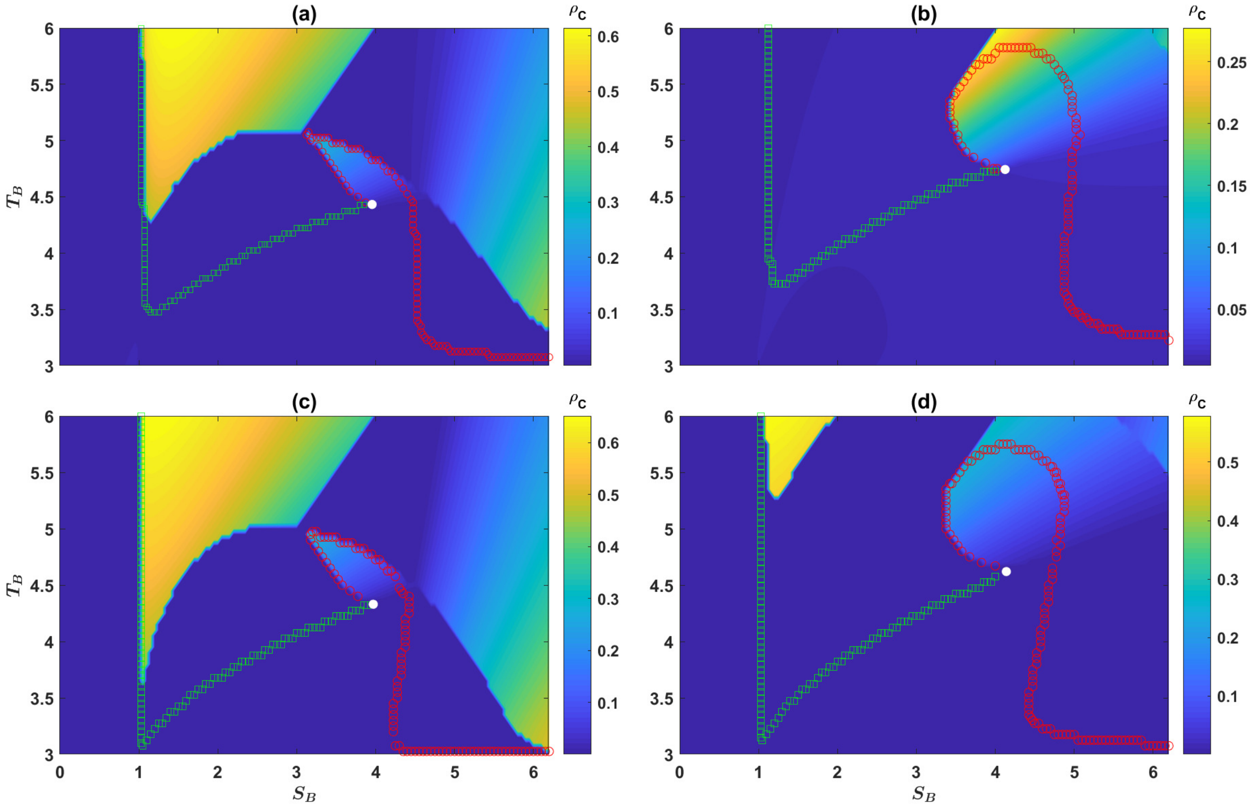

Figure Q: The effect of mutation rate in a mixed population. The color plot of the density of co-operators, in the direct interaction model (a) and (c), and the reputation-based model (b) and (d), in the $S_B - T_B$ plane. In (a) and (b) $\nu = 0.005$, and in (c) and (d) $\nu = 0.0005$. The boundaries of bistability are plotted as well. Below this boundary the dynamic is mono-stable, settling into a defective fixed point with a low level of cooperation. Above the boundary, a cooperative fixed point becomes stable and the dynamics become bistable. The two branches of the boundary meet at a critical point, where the transition becomes continuous. Comparison shows cooperation evolves in a broader range of parameter values for smaller mutation rate. Here, the replicator dynamics is solved starting from an unbiased initial condition in which the density of all the strategies are equal. We have set $R = 3$, $S = 0$, $P = 1$, $T = 5$, $R_B = 3$, and $P_B = 1$. In (b) and (d) $\eta = 0.1$.

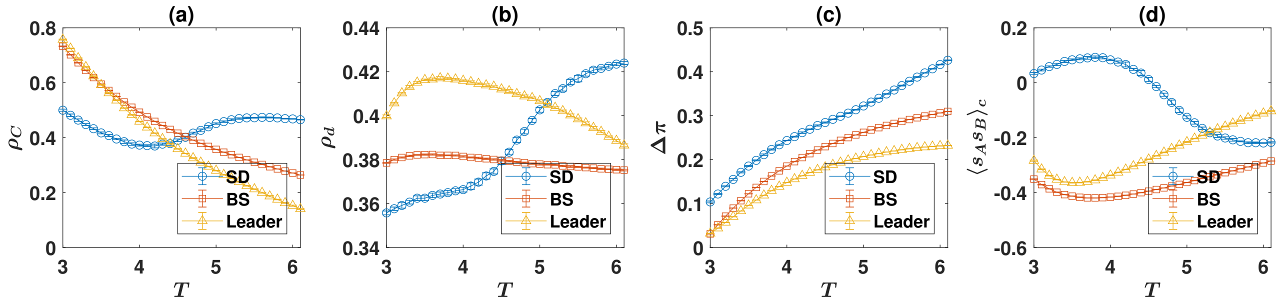

Figure R: The direct interaction model with three archetypal games in a structured population. The density of cooperators (a), the density of soft strategies in game B (b), the normalized payoff difference of cooperators and defectors in game B (c), and the correlation between the strategy of the individuals in the two games (d), as a function of the temptation, T, are plotted. The payoff values used for the games are presented in Table. B. Simulations are performed in a population of size 40000 individuals residing on a 200×200 square lattice with first nearest neighbor von Neumann connectivity and periodic boundaries. The simulations are performed for 6000 time steps, and averages and standard deviations are calculated based on the last 4000 time steps. The simulations start from random initial conditions. Here, $\nu = 0.005$.

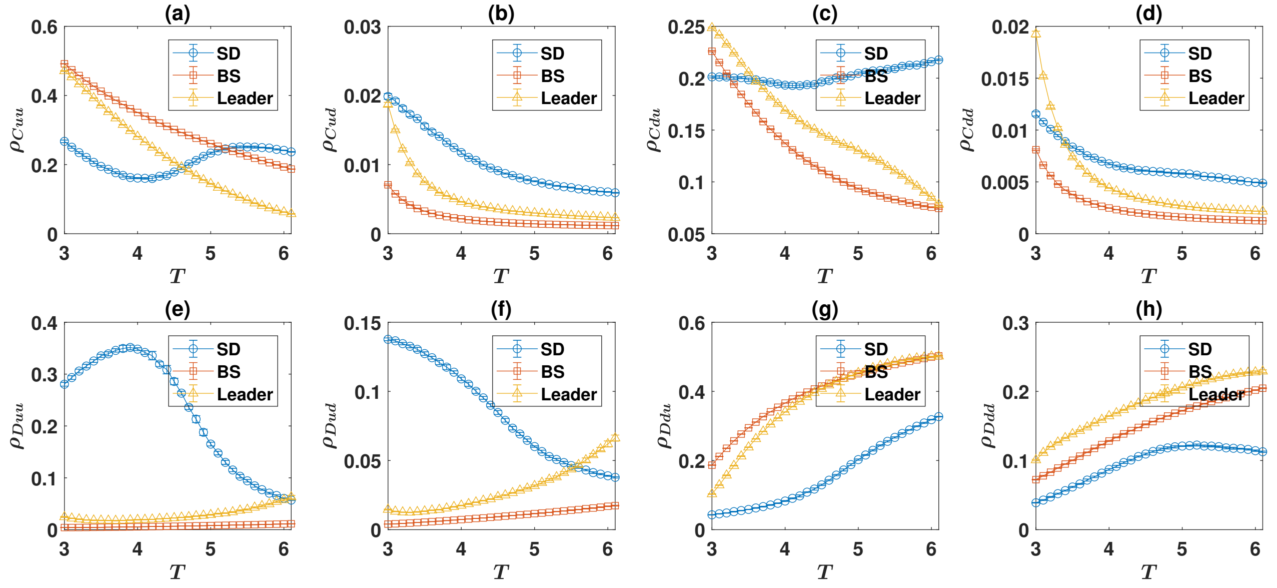

Figure S: The density of different strategies in the direct interaction model with three archetypal games in a structured population. The time average density of different strategies as a function of the temptation, T, are plotted. The payoff values used for the games are presented in Table. B. Simulations are performed in a population of size 40000 individuals residing on a 200×200 square lattice with first nearest neighbor von Neumann connectivity and periodic boundaries. The simulations are performed for 6000 time steps, and averages and standard deviations are calculated based on the last 4000 time steps. The simulations start from random initial conditions. Here, $\nu = 0.005$.

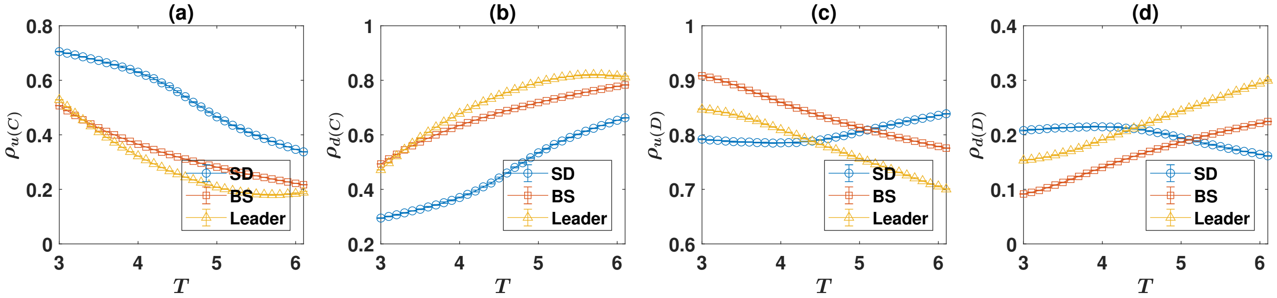

Figure T: Strategic response to cooperators and defectors in game B, in the direct interaction model with three archetypal games in a structured population. The time average density of strategies who play up with cooperators $u(C)$, down with cooperators $d(C)$, up with defectors, $u(D)$, and down with defectors, $d(D)$, as a function of the probability of error in inferring the PD strategy of the opponent, η, are plotted. The payoff values used for the games are presented in Table. B. Simulations are performed in a population of size 40000 individuals residing on a 200×200 square lattice with first nearest neighbor von Neumann connectivity and periodic boundary. The simulations are performed for 6000 time steps, and averages and standard deviations are calculated based on the last 4000 time steps. The simulations start from random initial conditions. Here, $\nu = 0.005$.

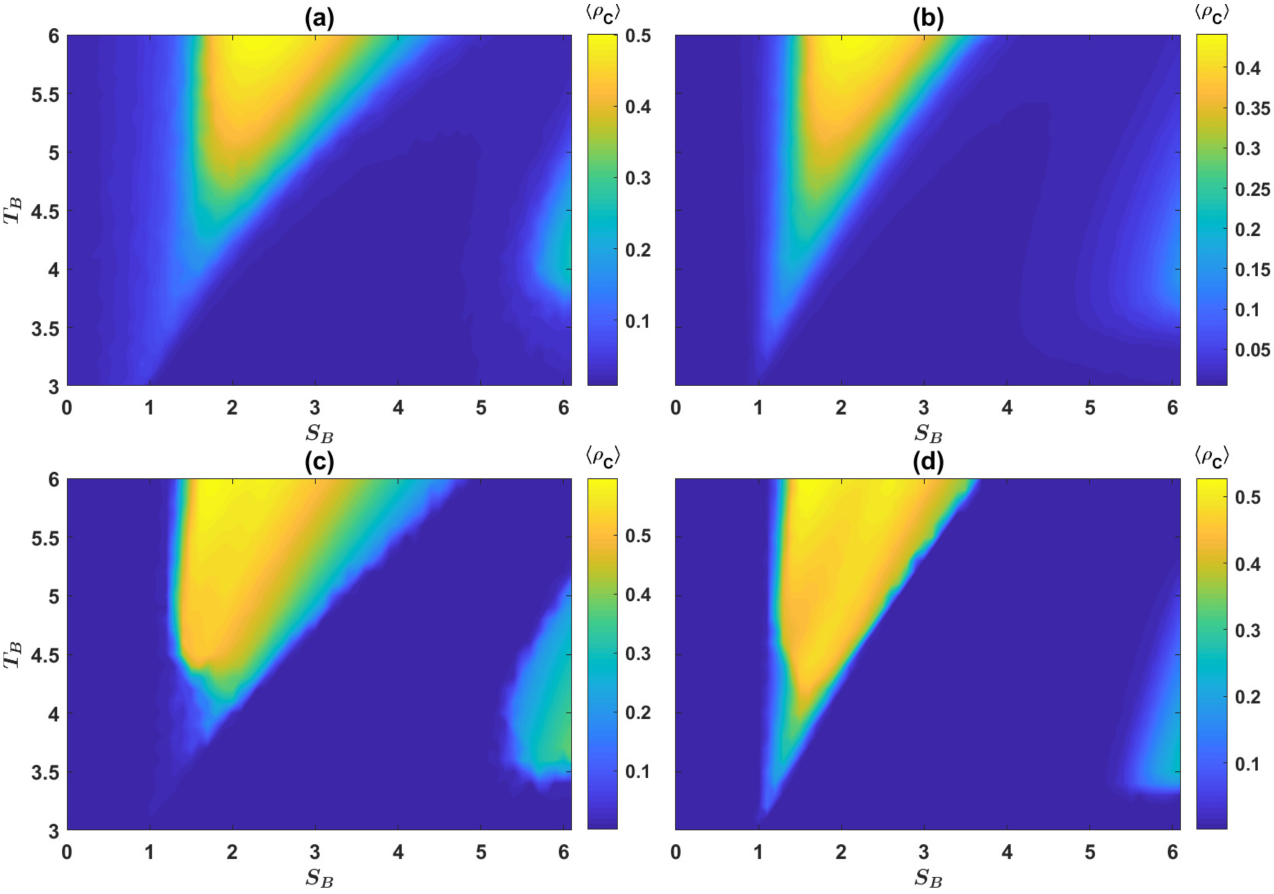

Figure U: The behavior of the models under continuous variation of the structure of game B in a structured population and for different mutation rates. The color plot of the density of cooperators, in the direct interaction model (a) and (c), and the reputation-based model (b) and (d), in the $S_B - T_B$ plane. In (a) and (c), $\nu = 0.005$, and in (b) and (d) $\nu = 0.0005$. Here, $R = 3$, $S = 0$, $P = 1$, $T = 5$, $R_B = 3$, and $P_B = 1$. The simulations are performed in a population of size 40000 individuals residing on a 200×200 first nearest neighbor square lattice with periodic boundaries and von Neumann connectivity. In (b) and (d) $q = 0.1$. In all the cases an unbiased initial condition (random assignment of strategies) is used.
