# Supplementary material for: Interaction between games give rise to the evolution of moral norms of cooperation
Source: PLoS Comput Biol. 2022 Sep 29;18(9):e1010429. doi: 10.1371/journal.pcbi.1010429 (PMC9521931; doi:10.1371/journal.pcbi.1010429)
Supplement: Attachment — Submitted filename: response.pdf [file pcbi.1010429.s005.pdf]

# Responses to review comments

## Response to Reviewer N. 1

**Reviewer N. 1:**
Reviewer N. 1: This manuscript theoretically explores an emergent mechanism of cooperation that supports moral norms by using both analytical solutions using replicator dynamics and individual-based simulations. In a prisoner's dilemma game and subsequent asymmetric Nash game, they show that a kind of payoff adjustment mechanism works by deciding the strategy of the subsequent game according to the strategy of the PD game, and as a result, a cooperative regime emerge.

This manuscript has sufficient originality and detailed analysis and may reach the publication level of PlosCB. For that purpose, it is necessary to reorganize the treatise so that it is easy for the readers to understand, mainly from the viewpoint of ingenuity of presentation. There are other improvements as well.

**Response:**
I am thankful to the reviewer for his/her careful reading and assessment of the manuscript, for his/her constructive suggestions, and for finding the manuscript of interest.

**Reviewer N. 1:**
1) I think that a phase diagram towards cooperation is summarized as follows: Rare $C-$ $[*s*]$ predominates$->$ phase transition$->$ $[C*h]$ and $[Ds*]$ dominate. Is my understanding correct? Anyway, the additional conceptual diagrams that depict the mechanism will help audience to understand clearly.

**Response:**
Yes, this understanding is correct. In the revision, I have added a figure, Fig. 3, where a Pi chart of different fixed points are presented. From this, it should be now graphically clear that what strategies exist in each phase.

**Reviewer N. 1:**
2) On lines 243-246, they claim that an asymmetric Nash, not a PD, is a necessary condition, but there is no evidence there. The reader should wait from line 301 to line 310 while maintaining frustration. Furthermore, the discussions on lines 180-184 and 248-251 are very important, but the conclusion is placed on lines 257-277. A well-streamed presentation requires a restructuring of the logic.

**Response:**
The organization of the paper is significantly modified. The revised manuscript begins by an static analysis of the games and proceeds step by step to uncover the phenomenology and underlying mechanism. I hope that the revised version has a much better organization.

**Reviewer N. 1:**
3) The captions in Figures 1 and 2 and the main text are redundant. Moreover, the captions themselves are not enough to fully understand those figures. For example, I don't know that DeltaPai indicates whether C or D is dominant. There is a typo in the definition of DeltaPai on line 177.

**Response:**
The captions are revised to better reflect the main message of the figures. The redundancy of caption of Figure 1 (Figure 6 in the new version) with the main text is removed. The typo in the definition of $\Delta\pi$ is corrected.

**Reviewer N. 1:**
4) You need to correct the variable mismatch. which one is correct? d, u (line 88, Figure 7, Figure 8

and results section) or s, h (Figure 3). You have to unify them. In addition, they mention "R = 128 and T = 10000" on line 286, where R and T are payoff parameters. The same variable name should be avoided.

**Response:**

This variation in the name of strategies is intentional, not a mismatch. s and h stand, respectively, for soft and hard strategies. Depending on the payoff values, the soft strategy (s) can be d or u. For most of the text (base payoff values) s is chosen to coincide to d and h to u. But when the continuous variations of the structures of the games is considered (Figure 8), s can either coincide with d or u, depending on what region of the phase diagram we are in.

In the revised version, $T$ and $R$ are only used for the payoff parameters of the games.

**Reviewer N. 1:**

5) When I read line 172, I feel that the author implicitly thinks "soft strategy = cooperation". Is that so?

**Response:**

Soft strategies can be considered as a form of cooperation, but a less self-sacrificing and more self-centered form of cooperation compared to cooperation in the PD, as they can naturally occur in the Nash equilibrium (such as the down strategy in the SD). I have added a note to this place to clarify this point (line 468-475 in the revision). I have tried to reserve the term cooperation for the cooperation in the PD, and use cooperative behavior to also refer to soft strategies.

## Response to Reviewer N. 2

**Reviewer N. 2:**

In this paper, the author explores the evolutionary dynamics that arises when players sequentially engage in two games. The first game is assumed to be a prisoner's dilemma. The second game is an anti-coordination game with asymmetric equilibria (such as the snowdrift game). Importantly, behavior in the second game may depend on the co-player's behavior in the first game (e.g., a player may reward co-players who cooperated in the first game by coordinating on their preferred equilibrium in the second game).

The author explores several variants of this model, a) depending on whether or not the same players engage in the two games ("direct interaction" vs "reputation-based" model), and b) depending on whether the population is well-mixed or whether games take place on a two-dimensional lattice.

The paper reports a two-fold advantage of coupling the two games: a) the coupling makes it more likely that players cooperate in the prisoner's dilemma b) the coupling makes it easier to coordinate on an equilibrium of the second game

(B) Overall assessment

In my opinion, the considered setup is quite interesting and understudied. The relevant question is: Even if we know evolution in each of simple normal-form games, which further effects can arise when two or more of these games are coupled in a non-trivial way. The current article gives some interesting answers. It shows that such a coupling can simultaneously enhance cooperation in a prisoner's dilemma and coordination in the snowdrift game.

In addition, I'd like to positively mention that the author has obviously put quite some effort into the analysis of this framework. The results are shown for both well-mixed and structure populations, and in a setup where players interact directly in the two games, or indirectly. In some sense, there are sufficiently many results in this paper to provide material for two papers.

Having said that, I need to admit I found it rather difficult from reading the paper to really understand which mechanism is driving the presented results. I believe there are two reasons why I found it difficult: a) The results are entirely numerical (either solutions of the replicator equation are

plotted, or simulation results). b) Especially in the beginning of the results section, the author's pace is too quick. As such, the results too often have a "black box" feeling – the final results are reported but the underlying mechanisms that drive these results remain somewhat unclear. As a result, I'm not convinced that all the conclusions that the author draws are actually confirmed by the model.

Overall, I believe that the above weaknesses can be addressed, but it will require quite some additional effort. I give some more specific suggestions below. I am aware that this additional work makes the paper even longer; but I really believe this effort would help readers considerably to make sense of some of the results.

If the author is able to describe more clearly which mechanisms drive the observed results, I believe the paper is appropriate for PLoS Computational Biology.

**Response:**

I am thankful to the reviewer for his/her careful reading and assessment of the manuscript, for his/her constructive suggestions, and for finding the manuscript of interest.

**Reviewer N. 2:**

(C) Specific comments

(C.1) The paper would greatly benefit from a clear and detailed analysis of a simple baseline case, before even going into the evolutionary analysis. For example, the author could take the case of a well-mixed population, in the direct-interaction setup, where players play the standard repeated prisoner's dilemma in the first round (with R=3, S=0, T=5, P=1) and the snowdrift game in the second round (with R=3, S=1, T=5, P=0). The author could use this example to clearly describe the 8 possible strategies. Then the author could even display the entire 8x8 payoff matrix. To explain the basic logic of the game, the author could then provide a static equilibrium analysis of this baseline game. For example, what is the set of all subgame perfect equilibria (or alternatively, of all Nash equilibria) of this game? For those equilibria that lead to at least some cooperation in the first game, please describe the logic why cooperation in the first game can be stable.

This static analysis would help in several ways: a) It provides some intuition for the subsequent evolutionary analysis b) It immediately explains the fixed points of replicator dynamics (since any Nash or subgame perfect equilibrium is a fixed point of replicator dynamics) c) It makes the reader more familiar with the setup before attempting to understand the effect of various parameter changes (note that in the present version, already the very first figure shows the non-trivial effects of changing the temptation payoff in the prisoner's dilemma).

I am not sure how difficult it would be to offer a similar static analysis for the general case (where the first game is an arbitrary prisoner's dilemma and the second game is an arbitrary anti-coordination game). If possible, this would be a useful addition to the SI. It would help to explain how the feasibility of cooperation in the prisoner's dilemma depends on the payoff parameters. However, it might well be that such a general analysis is too difficult to accomplish, and I don't insist on it.

**Response:**

I thank the reviewer for this insightful suggestion. I have added a section to the manuscript for static analysis. Both Snow Drift and Stag Hunts are studied and pure strategy Nash equilibria are derived. Simple conditions for the existence of a cooperative equilibria are derived. Mixed strategy equilibria correspondings to the fixed points of the dynamics are derived in a rather general case, from which simple conditions for the existence of a cooperative fixed point for the Stag Hunt game is derived.

**Reviewer N. 2:**

(C.2) Related to the previous point, the author mentions at several places that for cooperation in the prisoner's dilemma to be feasible, the second game needs to have an asymmetric equilibrium. I'm not convinced this is true. To see why, assume that the second game is a coordination game, like Stag-Hunt, with payoffs R=5, S=0, T=1, P=1. Consider the following strategy: Cooperate in the prisoner's dilemma; then play Stag (i.e., the more efficient equilibrium) if both players cooperated;

otherwise play Hare (the less efficient equilibrium). Then one can show: a) if both players adopt this strategy, they both cooperate in the prisoner's dilemma b) it is a subgame perfect equilibrium (and hence a fixed point of replicator dynamics).

Of course, the above strategy requires that players can condition their second-game behavior on the previous behavior of both players. However, it seems to me this example suggests that it's not the asymmetry of equilibria that is necessary for the results. Rather it is the fact that the second game needs to have more than one equilibrium (symmetric or asymmetric), and one of the equilibria is more profitable to a player than the other. I would thus like the author to discuss the case of a stag-hunt game as the second game more specifically.

**Response:**

I thank the reviewer for this constructive point. The Stag Hunt game is discussed in detail. It turns out the the reviewer is correct. cooperative eqiulibria for the Stag Hunt game exist in the mixed population. However, the underlying mechanism are different to those in the anti-coordination game. Furthermore, the basin of attraction of coordination games are much smaller, and these fixed points do not exist in a structured population. A detailed discussion of coordination games is added and mentioned in the abstract, introduction, results, and discussion.

**Reviewer N. 2:**

(C.3) One thing that I find hard to understand is the case of T=3 in Figure 1 for the snowdrift game. In this case, all of the players seem to cooperate in the prisoner's dilemma. However, if everyone cooperates in the prisoner's dilemma, it is no longer possible to use the first-game behavior as an effective coordination device for the second game. However, if the first round behavior does not help coordination in the second game any longer, why is this equilibrium routinely selected by individual based simulations? Could the author explain this case in more detail?

**Response:**

The dynamical mechanism for $T = 3$ is similar to other cases, shown in Fig. 3. As even for T=R (T=3), defection dominates cooperation in the prisoner's dilemma, defectors would grow and dominate the population in a simple prisoner's dilemma. This also happens in the model in the beginning of the time evolution. Once defectors increase in numebr, playing soft with cooperators incurs a low cost. Thus strategies that play soft with cooperators increase faster than those who play soft with defectors. This leads to a rapid dynamical transition to a state where the cooperation favoring norm of playing soft with cooperators and cooperation dominate. Once this happens, the dynamics get fixated into the cooperative state where cooperators compose a large fraction of the population. In other words, fixation of cooperation-favoring norms results from low frequency of cooperators during the transient dynamic of the system before stationary state is reached. I have clarified this point in the revised version, in the result, and mentioned in the discussion.

Also, using static analysis, I have shown that a fully cooperative equilibrium, corresponding to this regime, exist when game B is the Snow Drift game.

**Reviewer N. 2:**

(D) Minor comments

(D.1) It seems that in each case, the author only looks at one specific trajectory of replicator dynamics: the initial population is always assumed to consist in equal proportions of all players. This may appear "fair", but as a method to analyse a dynamical system it is rather unorthodox. When analysing such systems, I'd rather try to first describe all stable and unstable fixed points. In a second step, I'd then try to numerically estimate the basin of attraction of each fixed point. I don't think this is an important point, because the author's simulations are done for random initial populations (not just the perfect initial population that consists in equal proportion of all available strategies). However, I think it would be helpful to make it more explicit that only one single trajectory of replicator dynamics has been studied. In addition, I would avoid terminology like "equilibrium fixed

point" and "non-equilibrium fixed point" (as in lines 208-209). There is nothing special about the fixed point that is reached from a uniform initial distribution of strategies.

**Response:**

I thank the reviewer for this observation. In the revision, I have added result for solutions of the replicator dynamics starting from different initial conditions (Fig. 4). This, in addition to giving a measure of the basin of attraction of different fixed points, reveals a difference in the basin of attraction for the anti-coordination games and the Stag-Hunt game.

I have kept the equilibrium/non-equilibrium terminology. As this terminology is rooted in statistical physics, where the equilibrium state is defined as the state reaching from an unbiased initial condition, I hope using it in this context would be harmless. It also provides a simple way of labeling different fixed points.

**Reviewer N. 2:**

(D.2) Related to the previous point, when simulations and the solution according to replicator dynamics disagree, the author often speaks of a "finite size effect". However, given that the population size in most simulations is pretty large, I'm not convinced it's indeed the finiteness of the population that makes the difference. Rather I could well imagine that the difference is due to the fact that simulations are really based on random initial populations, whereas the author only studies replicator dynamics for a very specific initial population.

**Response:**

This can be part of the reason. However, I note that as initial strategies are assigned at random in simulations, in an infinite size population the frequency of all strategies would be equal. Only in a finite populations, there are deviations from the expected value (of order of $\sqrt{N}/N$). Thus, it seems it is safe to consider this as part of the finite size effects.

I also note that finite size effects are particularly strong in this model. The reason is that the time evolution has two phases. Initially the dynamics goes to a state where the frequency of strategies are close to the defective fixed point. In the case of defection favoring initial conditions the dynamics eventually settles in the defective fixed point. In the case of cooperation favoring initial conditions, only after a long transient a rapid transition to the cooperative fixed point occurs from such a transient state. Random population fluctuations resulting from finite size effects can drive dynamical transition to the cooperative fixed point, even for some initial population configurations where for infinite population the dynamics settle in the defective fixed point. Intuitively, the strong finite size effects come from the fact that some strategies are neutral. For instance, in a population composed of defectors, those strategies which play soft or hard with cooperators receive the same payoff due to the absence of cooperators (that is e.g., $Ddu$ and $Duu$) and are subject to random drift in a finite population. The size of these drifts is larger, smaller the population. Sometimes cooperation favoring ones build-up enough to initiate a dynamical transition to the cooperative fixed point.

**Reviewer N. 2:**

(D.3) Line 56: At this point of the text, it is difficult to understand what it means to "play softly with cooperators", because the meaning of "soft" hasn't been explained yet. Maybe it's better to just say "play the strategy that gives a higher payoff to cooperators"

**Response:**

I have added an explanation to this part.

**Reviewer N. 2:**

(D.4) Line 124: I believe the appropriate condition should read "T+S ¡ 2R" instead of "T¡2R" (note that this is inconsequential, because S is set to zero for this prisoner's dilemma).

**Response:**

I have corrected this error.

**Reviewer N. 2:**

(D.5) Figures 1-2: It would be very helpful if each row had a header saying "snowdrift game", "battle of sexes" and "leader", respectively. Also, it would be useful if the y-axis ranges from -0.05 to 1.05 [instead of 0 to 1], such that one can better see fixed points that are exactly on the boundary.

**Response:**

I have applied these changes.

**Reviewer N. 2:**

(D.6) In Figure 2, please say which value of T is used for this simulation (I assume T=5, but it is better to be sure). Also, I don't think the meaning of the variable nu has been explained at this point, so please write "Here, the mutation rate is nu=0.005" instead of "Here, nu=0.005".

**Response:**

As stated in the caption the base payoff values presented in Table 2 is used in which $T = 5$.

**Reviewer N. 2:**

(D.7) I don't think it is very useful to consider error-values larger than 0.5. An error-rate eta ¿ 0.5 just means that the labels "C" and "D" switch their meaning. For example, in the extreme case, eta=1, there are basically no errors at all, just players start calling each C of the co-player "D". Of course, the author is aware of this, and the results are explained appropriately. But still, I think the paper would become clearer if the error rate eta was restricted to be at most 0.5 from the outset (and to only show the left half of each panel in Figure 2, for example).

**Response:**

This point is true. However, I have chosen to keep errors larger that 0.5 in the figures, as it appears to me the plot are more appealing in this way. One reason is that in this way optimization of cooperation in the Stag Hunt game in structured populations is pronounced. Furthermore, the symmetry of the plots are an assuring reminder of the consistency of the results.

**Reviewer N. 2:**

(D.8) In line 312-316, the author argues that in typical models of structured populations, network reciprocity can only promote cooperation if the individuals are selected for reproduction with a probability proportional to the exponential of their payoff. I don't think this statement is true (and the author does not provide any reference that shows that this exponential form is indeed necessary).

**Response:**

I have removed this part. However, I am pretty confident that this is true. All the models that I am aware of use this exponential form. My investigation also shows cooperation does not evolve in a simple PD using a linear form (of course it can evolve with a linear form if other auxiliary mechanisms are at work, e.g., Salahshour, M., 2021. Evolution of cooperation in costly institutions exhibits Red Queen and Black Queen dynamics in heterogeneous public goods. Communications biology, 4(1), pp.1-10.). For instance, in the new figure, Fig. 9, where game $B$ is a Stag Hunt game, it can be seen that cooperation does not evolve. But, momentarily, I do not remember of a reference which explicitly state this. Even for an exponential form, there are phase transitions in the system such that cooperation evolve for some range of the selection parameter $\beta$ (and in general other parameters such as cost and benefit of cooperation ref (Szab, G. and Fath, G., 2007. Evolutionary games on graphs. Physics reports, 446(4-6), pp.97-216.)).

**Reviewer N. 2:**

(D.9) Lines 397, 400: "see of Duds" should probably read "sea of Duds"

**Response:**

This typo is corrected.

**Reviewer N. 2:**

(D.10) It is somewhat confusing that in the SI page 2, the first strategy is called "down" and the second is called "up". Usually it's the other way round.

**Response:**

As $d$ is in a sense a cooperative strategy, in the table I have shown it as the first one to conform with the PD. In other places, I use a consistent numeration of all the possible strategies.

## Response to Reviewer N. 3

**Reviewer N. 3:**
Reviewer 3: In this article, the author uses numerical simulations to study a model in which individuals first make a choice in a Prisoners dilemma and then make a choice in a game B. Reputation is formalised through a probability of guessing the choice of the partner (in the B game) in the previous PD, before playing B. It is argued that, whenever B has a non-symmetric equilibrium, cooperation may evolve in this model, both in structured and mixed populations. I think that the result is overstated. The model is tested only in some particular games B, therefore it is impossible to conclude that the results hold for every game B with a non-symmetric equilibrium. Moreover, the paper is missing a theoretical analysis. My feeling is that, whenever B has an equilibrium in mixed strategies, then cooperation in PD might be supported in equilibrium of the sequence of games, and thus one might have the evolution of cooperation. So, my feeling is that the paper fails to convince the reader that the issue preventing the evolution of cooperation is really the symmetry of the Nash equilibrium of B, or rather is the support of the equilibria.
Another limitation is the lack of motivation: what are real life examples of B? Why shall we care about this method to promote cooperation? What is the biological relevance of this model? In sum, I do think that this paper has the potential to make a valuable contribution, but, at this stage, it is at a too early stage to be considered for publication, especially in a highly selective journal such as Plos CB.
**Response:**
I hope the revision provides a better answer to these questions. In the revision it is shown that cooperation can evolve when game B is a coordination or anti-coordination game. However, the underlying mechanism and basin of attraction are different for the two cases. Theoretical analysis, including conditions for the existence of a cooperative Nash equilibrium and fixed point is added, following insightful suggestions from reviewer 2. In this regard, important results regarding the existence of a cooperative equilibrium is added. Biological or social relevance is better discussed, new references to instances that the mechanisms suggested here can be at work (such as evolution of costly signals, and harmful norms), and new citation to the literature are added.
