# Supplementary material for: Interaction between games give rise to the evolution of moral norms of cooperation
Source: PLoS Comput Biol. 2022 Sep 29;18(9):e1010429. doi: 10.1371/journal.pcbi.1010429 (PMC9521931; doi:10.1371/journal.pcbi.1010429)
Supplement: Attachment — Submitted filename: response.pdf [file pcbi.1010429.s006.pdf]

Responses to review comments

Summary of changes

The manuscript is revised to improve the clarity of the presentation. A paragraph is added to the end of the introduction as a guide to the structure of the article. Wherever it was judged to be helpful, a summary of the content of each subsection is added to the beginning of the subsections. The language and presentation of the manuscript is edited based on referee two's comments.

Response to Reviewer N. 1

Reviewer N. 1:
I have confirmed that the revised manuscript has been significantly improved in response to all comments including myself, and thus I recommend the publication.
Response:
I would like to thank the reviewer once again for his/her careful reading and assessment of the manuscript and for his/her constructive comments.

Response to Reviewer N. 2

Reviewer N. 2:
GENERAL EVALUATION.

The paper explores what happens if individuals engage in two consecutive games. The first game is a social dilemma (prisoner's dilemma); the second game is an anti-coordination or coordination game (e.g., a snowdrift game or a stag-hunt game). The author shows that this coupling of games can have two benefits:
(1) It can increase cooperation in the first game
(2) It can avoid coordination failures in the second game

Already in my first report, I argued that the general setup is interesting and worth studying. Also, I argued that the author has put extensive work into his analysis, by exploring various different scenarios (e.g., varying the exact anti-coordination game to be played as the second game; exploring both direct and indirect interaction scenarios; comparing well-mixed and lattice-based populations; replicator dynamics and simulations, etc).

On the downside, I argued that it is sometimes hard to understand the results, because they often feel like coming out of a black box. To address these weaknesses, I suggested various clarifications. For example, I suggested that the author offers an explicit (Nash) equilibrium analysis. Also, I suggested that the author also considers the case in which the second game is a coordination game, rather than an anti-coordination game. The author has taken all these suggestions into account. I believe the intuition for what drives the observed effects is now clearer, and I would like to thank the author for his efforts.

In some sense, I believe the manuscript contains actually too much information for a typical journal article. By considering so many distinct scenarios, it is somewhat difficult for the reader to fully grasp the nature of all results. I believe it would have been useful to write a first paper only about the baseline case, direct interactions in well-mixed populations. This case could then have been analysed in full detail, and the results could have been explained more clearly.

Having said that, I believe also the current article is fine. In the following, I only make a few minor suggestions to increase the clarity of the text.

Response:

I would like to thank the reviewer once again for his/her careful reading and assessment of the manuscript and for his/her constructive comments.

Reviewer N. 2:

COMMENTS:

[Line and page number references refer to the version with annotated changes]

(1) In the abstract, the author claims that "coupling between the two games emerges and helps to solve the dilemma" I would prefer if the author were more careful here. Coupling does not fully solve the dilemma (as it typically does not lead to full cooperation in the prisoner's dilemma, but only say 60%). Please describe more clearly that coupling may promote cooperation; however, at least if the second game is an anti-coordination game, full cooperation in the first game still requires additional mechanisms. Moreover, more cooperation in the first game might make it more difficult to anti-coordinate in the second game.

Response:

I have modified the abstract to mention that coupling between games sustain cooperation in the social dilemma and has removed the claim of solving the social dilemma. This seems more faithful to the phenomenology presented in the paper. As a detailed account of the cooperation level can make the abstract even longer than it already is, and also as these detailed information might not be of out-most importance, I have added necessary modifications to clarify this points to the introduction and results.

Reviewer N. 2:

(2) Similarly, in the introduction it would be useful if the author more clearly explains what a "cooperative equilibrium" is. These equilibria are typically not fully cooperative; they only exhibit *some* cooperation in equilibrium.

Response:

I have added an explanation to clarify that often the equilibrium is not fully cooperative, but depending on the strength of the social dilemma and the structure of game B full cooperation is also possible.

Reviewer N. 2:

(3) At the end, of the introduction, the author states: "In contrast, when game B is a coordination game, moral norm of cooperation does not evolve in a structured population." I would prefer if the author chooses a more humble formulation, such as: "In contrast, in those simulations in which game B is a coordination game, we did not observe the evolution of cooperation." The reason why I suggest this is the following: the author's claim might be true for the simulations he performed. However, I could imagine several small modifications of the model such that cooperation might actually evolve even if the second game is a coordination game. For example, the author could have allowed for rare implementation errors in the first game, such that cooperators defect with some small probability epsilon. In that case, Cdu becomes a strict Nash equilibrium. Hence Cdu might also evolve in structured populations.

Response:

I have modified this part accordingly.

Reviewer N. 2:

(4) On page 5, line 150, I wouldn't motivate 1-eta as the likelihood of players "guessing" their co-player's previous action. Rather, I would say it is the likelihood that players are correctly informed about their co-player's previous action.

Response:

I have modified this part accordingly.

Reviewer N. 2:

(5) It would be good if the author explains more clearly early on which default payoff values are used for the different games. For example, in Table 1, it would be useful to see the base game payoffs at least in the figure caption.

Response:

I have moved table 1 summarizing the base payoff values to the Model Section where payoff parameters and game B is defined.

Reviewer N. 2:

(6) In Figure 2b, the author suggests that cooperation can emerge if the cost of cooperation is smaller than the "cost of coordination failure". However, game theorists usually do not interpret $R_B - P_P$ as the cost of coordination failure in a stag-hunt game (rather coordination failure would be typically associated with the risk of getting one of the non-equilibrium payoffs T_B or S_B). Rather $R_B - P_B$ is the cost of coordinating on the payoff-dominated equilibrium.

Response:

I have changed the terminology in the figure and in the text based on this suggestion.

Reviewer N. 2:

(7) In Figure 3, are these all possible fixed points, or is it just a selection? Also, it would be useful if the author always speaks of the replicator-mutator dynamics (as standard replicator dynamics wouldn't allow for mutations). In addition, it would be useful if the author points out that he uses a discrete-time replicator dynamics (because I'd assume most readers would associate replicator dynamics with the continuous version).

Response:

These are the stable fixed points that I detected by solving the replicator dynamics for different initial conditions. Although I have solved the replicator dynamics for a large set of different initial conditions (random or special initial conditions, in which e.g. only some of the strategies are found in non-zero frequency, or initial condition corresponding to the Nash equilibria of the static games), and no other fixed points was observed, it can not be claimed that this is an exhaustive set of fixed points.

Reviewer N. 2:

(8) On page 8, the author uses formulations like fixed point "SH1". Please define SH1 at its first occurrence.

Response:

I have added a definition of these fixed points on their first appearance in page 8.

Reviewer N. 2:

(9) The caption of Figure 5 does not match the figure (panel a seems to have switched places with panels b and c).

Response:

I have corrected the caption to match the figure.

Reviewer N. 2:

(10) Line 417; please correct "$p_C^{initial}$.

Response:

This is corrected.

Reviewer N. 2:

(11) When using an initial condition in which all strategies are played with equal frequency, please do not speak of a "random initial population". This initial population is not randomly chosen, it is a very specific initial population. Instead one may speak of the "center" initial population.

Response:

This modification is applied in the revision.

Reviewer N. 2:
(12) On page 18, I would be careful attributing the differences between simulations and replicator dynamics only to "finite size effects". After all, the simulations differ from replicator dynamics in more than just the finite size of the population. It is not clear that it is the finite size of the population that is responsible for the differences.
Response:
I have added an explanation that one possible mechanism for finite size effect to be at work is population noise resulting from finite size of the population giving rise to deviation of the initial condition from the center initial conditions used in solving the replicator dynamics.

Reviewer N. 2:
(13) Please do not speak of "equilibrium fixed points" and "non-equilibrium fixed points". After all, both types of fixed points correspond to Nash equilibria. It is somewhat strange to call the second Nash equilibrium a "non-equilibrium fixed point". Please find a different name to refer to the two fixed points.
Response:
I have replaced equilibrium and non-equilibrium fixed points with, respectively, unbiased and biased fixed points.
Reviewer N. 2:
(14) In general, it would be helpful if the axis labels of the figures do not only mention the variable names (like "eta" or "rho_C"), but also some text that describes this variable (like "Error rate" or "Probability to cooperate in first game"). It is hard for a reader to keep all variables in memory, so any help here is appreciated.
Response:
In places where such an explanations were missing, I have added a clear definition of symbols in the caption to help the understanding. I have chosen to keep symbolic notations for the axis labels to keep all the figures consistent and keep the notation brief.
Reviewer N. 2:
(15) Please avoid referring to second game behavior as "cooperation" (as on page 23, line 739). The text becomes clearer if only first game behavior is interpreted as cooperation.
Response:
I have reserved the term "cooperation" for cooperation in social dilemma and had occasionaly used cooperative strategies for game B, when suitable. However, following the reviewer's suggestions, I have removed references of cooperative strategies to strategies of game B and replaced it with soft strategies.
Reviewer N. 2:
(16) Page 25: There are multiple instances of "see of" which should read "sea of"
Response:
This is corrected.
Reviewer N. 2:
(17) In the discussion, I would prefer if the author rephrases the sentence "This appears to provide a possible explanation for the evolution of morality" Instead of "explanation", I would prefer "a possible mechanism". In my opinion, this would make more clear that the proposed model certainly does not explain all aspects of morality.
Response:
This modification is applied.
Reviewer N. 2:
(18) In the discussion, what exactly does the author mean when saying that "assessment module and

action module need to be placed on the same level"? Please rephrase. Also, please note that there are indirect reciprocity models where players only need first-order information (for example the models of Ulrich Berger, or the model of the recent PNAS paper of Drew Fudenberg and colleagues, on "simple records").

Response:

I have clarified the writing in this part.

Reviewer N. 2:

(19) As a general piece of advice, I think an article like this would be easier to read if the author engaged more in meta-communication with the reader. Like, in the beginning of a paragraph, make sure the reader understands what the paragraph will be about. At the end of the paragraph, make sure the reader understands what the take-home message is.

Response:

Following this comment, I have added a guide to the structure of the article at the end of introduction, and a motivation and summary of what is going to be done in each section or subsection to the beginning of the sections or subsection. I have also edited the language to improve the clarity of the presentation.

Response to Reviewer N. 3

Reviewer N. 3:

I plause the author for a very competent revision, which addressed all my comments. I think that the paper can be published. Just one minor comment, that the author may or may not follow: a review article on the role of moral norms on cooperation and other forms of pro-sociality has been recently published, I think that the author may find it interesting for his research:

https://royalsocietypublishing.org/doi/full/10.1098/rsif.2020.0880

Response:

I thank the reviewer for his/her positive assessment of the manuscript. This reference is added to the citations.
